# HIV-1 cell-to-cell infection of macrophages escapes restriction by the BST-2 host cell factor independently of the Vpu counteracting viral protein

**DOI:** 10.1371/journal.ppat.1014702

**Published:** 2026-10-23

**Authors:** Sen Yan, Agathe Durringer, Marie Woottum, Serge Benichou

**Affiliations:** 1 Institut Cochin, CNRS, Inserm, Université Paris Cité, Paris, France; 2 NABI Laboratory, CNRS, Inserm, Université Paris Cité, Paris, France; University of North Carolina at Chapel Hill, UNITED STATES OF AMERICA

## Abstract

As HIV-1 target cells, macrophages can be infected by cell-to-cell virus transfer from infected T cells through cell-cell fusion leading to the formation of virus-productive multinucleated giant cells (MGCs). We recently demonstrated that this route of infection escaped host cell restriction acting on the early steps of the viral life cycle. Here, we investigated whether restriction factors acting on late steps of HIV-1 replication were also bypassed in virus cell-to-cell infection of macrophages. We focused on the impact of the interferon(IFN)-induced factor BST-2/tetherin acting on virus release, a process counteracted by the Vpu viral protein. We show that deletion of Vpu did not significantly affect HIV-1 cell-to-cell infection of IFN-stimulated macrophages, and virus assembly in virus containing compartments was not affected in MGCs. Accordingly, depletion of BST-2 in IFN-stimulated macrophages had no impact on virus cell-to-cell transfer, formation of MGCs and virus production. However, cell-to-cell infection of macrophages was still sensitive to the nelfinavir protease inhibitor, whereas we previously showed that this route of infection was resistant to antiviral drugs acting on early steps of virus replication. Our results show that HIV-1 cell-to-cell infection of macrophages escapes restriction factors acting on both early and late steps of the virus life cycle, but displays resistance to antiviral drugs specifically acting on the early steps of virus replication.

## Author summary

Macrophages participate in HIV-1 dissemination, as well as in the establishment of the tissue virus-reservoirs. The work reported here show that HIV-1 cell-to-cell infection of macrophages from infected T cells, escapes restriction by the interferon-stimulated BST-2 protein acting in the last step of virus release. We demonstrate that this mechanism is independent of expression of the Vpu counteracting viral protein. However, in contrast to the resistance previously observed to antiviral drugs acting on early steps of virus replication, cell-to-cell HIV-1 spreading in macrophages remained sensitive to the nelfinavir protease antiretroviral inhibitor acting on virus maturation. These results contribute to a better understanding of HIV-1 persistence and evasion to host innate immune response in people living with HIV.

## Introduction

Infected myeloid cells, including macrophages, are found in many tissues of people living with HIV [1], often as infected multinucleated giant cells (MGCs). As HIV-1 target cells, they participate in all steps of HIV-1 infection, including virus transmission and dissemination, as well as in the establishment of tissue virus reservoirs, which represent a major obstacle to virus eradication and contribute to persistence of adverse effects in infected patients despite antiretroviral therapy [2–9].

At least *in vitro*, macrophages are difficult to infect by cell-free virus particles, and the first block in virus replication is related to virus entry and specific cellular tropism of the HIV-1 variants found in infected individuals and characterized as non-macrophage(non-M)-tropic in cell-free infection assays [10,11]. Moreover, the poor virus replication in macrophages is also largely related to the high expression of host cell restriction factors, often expressed through interferon stimulation, that inhibit specific steps of the viral life cycle. Inhibition of reverse transcription of the viral RNA in proviral DNA by the SAMHD1 cellular factor is certainly the main mechanism that limits efficient HIV-1 replication in macrophages. Like SAMHD1, most of the other known host restriction factors, such as members of the SERINC, IFITMs, TRIM and APOBEC3 protein families, also inhibit entry or post entry early steps of virus replication, before integration of the viral DNA into the host genome [12]. Interestingly, the BST-2/tetherin protein is the best characterized restriction factor acting on the late steps of the virus life cycle [13]. However, HIV-1 specifically expresses the auxiliary Vpu protein for counteraction of the inhibitory activity of BST-2 for most groups of HIV-1 strains, excepted group O strains that use the viral Nef protein for BST-2 counteraction and group P viruses that do not appear to have developed mechanisms to counteract BST-2 [13–15]. In macrophages, the late steps of virus assembly and the budding of the virus particles take place as usually at the cell surface, but also in a specialized intracellular membrane compartment called VCC, for virus-containing compartment, in continuity with the plasma membrane [16,17].

In addition to cell-free virus infection, it is now established that enveloped viruses such as HIV-1 can infect target cells by viral cell-to-cell transmission. This process involved direct contacts between virus-donor infected cells and recipient uninfected target cells [11,18–21]. This cell-to-cell virus transfer likely represents the dominant mode of virus dissemination *in vivo* in numerous lymphoid and non-lymphoid tissues [19,21,22]. We and others have reported that this route is very efficient for HIV-1 spreading in myeloid cells, such as macrophages, from infected virus-donor T cells [23–28]. Infected macrophages are detected in many tissues, including the central nervous system, lymph nodes, spleen, lungs, and digestive and genital tracts [1]. Remarkably, these infected myeloid cells are often found as MGCs [29–40]. In agreement, our works identified a new route for rapid HIV-1 cell-to-cell dissemination from infected CD4+ T cells toward myeloid cells, including macrophages, by a two-step cell-cell fusion mechanism [24,25]. In the first step, infected T cells establish contacts with macrophages, resulting in cell-cell fusion with these target cells. The newly formed HIV-1 Gag+ fused cells are then able to fuse with surrounding non-infected macrophages, leading to the formation of MGCs that can survive for more than 30 days in culture and produce high levels of *de novo* infectious virus particles [12,24,25,27,28].

Importantly, we have recently shown that this route of HIV-1 cell-cell infection of macrophages fusion escaped host cell restriction mediated by SAMHD1, SERINC5 and APOBEC3G proteins, acting on the early steps of the viral life cycle [25,28]. This virus-mediated cell-cell fusion is also largely resistant to antiviral drugs acting on the early steps of the virus life cycle, such as reverse-transcriptase, capsid and integrase inhibitors. The goal of the present study was to investigate whether the interferon-induced restriction factor BST-2 acting at the late steps of the viral life cycle, counteracted by the viral Vpu protein, is also bypassed when IFN-stimulated macrophages were infected by virus cell-to-cell transfer. In addition, we explored whether HIV-1 cell-to-cell infection of macrophages was still sensitive to the nelfinavir protease inhibitor. Together, our results show that HIV-1 cell-to-cell transmission to macrophages from infected T cells escapes restriction imposed by host IFN-stimulated cell factors acting on both early and late steps of the virus life cycle Interestingly while displaying resistance to antiviral drugs specifically acting on the early steps of virus replication, virus cell-to-cell spread remained sensitive to the nelfinavir virus maturation inhibitor.

## Results

### HIV-1 cell-to-cell infection of macrophages is independent of the Vpu viral protein

In order to investigate whether the viral Vpu auxiliary protein was able to modulate virus cell-to-cell transfer for infection of macrophages from infected CD4 T cells, we used CD4 primary T cells from blood of healthy donor serving as virus-donor cells and autologous primary monocyte-derived macrophages (MDMs) as target cells. T cells were infected with the wild-type (WT) or Vpu-deleted viruses (ΔVpu) of the macrophage-tropic NLAD8 viral strain and infection was evaluated by flow cytometry and Western blot analyses (Fig 1A and 1B). Infected T cells were then cocultured for 24 h with MDM targets as schematized in Fig 1C and described previously [24,27]. Infected CD4 T cells were eliminated by intensive washings before staining of the viral Gag precursor protein with a specific antibody in macrophage targets. The percentage of Gag+ MDMs was then analyzed by flow cytometry directly after coculture (Fig 1D) or after 6 additional days (Fig 1E) for evaluation of the rate of virus spreading as previously [25,27]. When analyzed for viral Gag expression in MDMs infected by cell-free viruses, very low virus transfer was detected in MDMs infected with high doses (250 ng of viral p24) of either WT or ΔVpu viruses after 24 h of infection (Fig 1D). In contrast, significative infection of macrophage targets around 10% was detected after 24 h of coculture with both WT- and ΔVpu-infected T cells viruses (Fig 1D). Notably, when analyzed 6 days after infection, no differences in macrophage infection by viral cell-to-cell coculture with infected T cells were observed when ΔVpu viruses were compared to WT viruses (Fig 1E). In contrast, expression of Vpu was largely required when macrophages were infected by cell-free viruses during 6 days (Fig 1F). This suggests that Vpu did not modulate virus cell-to-cell infection and dissemination compared to macrophages infected with cell-free viruses. In agreement, no differences were observed in virus production quantified by p24 ELISA in the supernatant of MDMs cocultured with WT- and ΔVpu-infected T cells (Fig 1G), in contrast to macrophage targets infected with cell-free viruses expressing WT viruses compared to macrophages infected withΔVpu viruses (Fig 1H). These results indicate that in contrast to cell-free virus infection, deletion of Vpu has no influence on HIV-1 cell-to-cell infection of macrophages from infected T cells.

**Fig 1:**
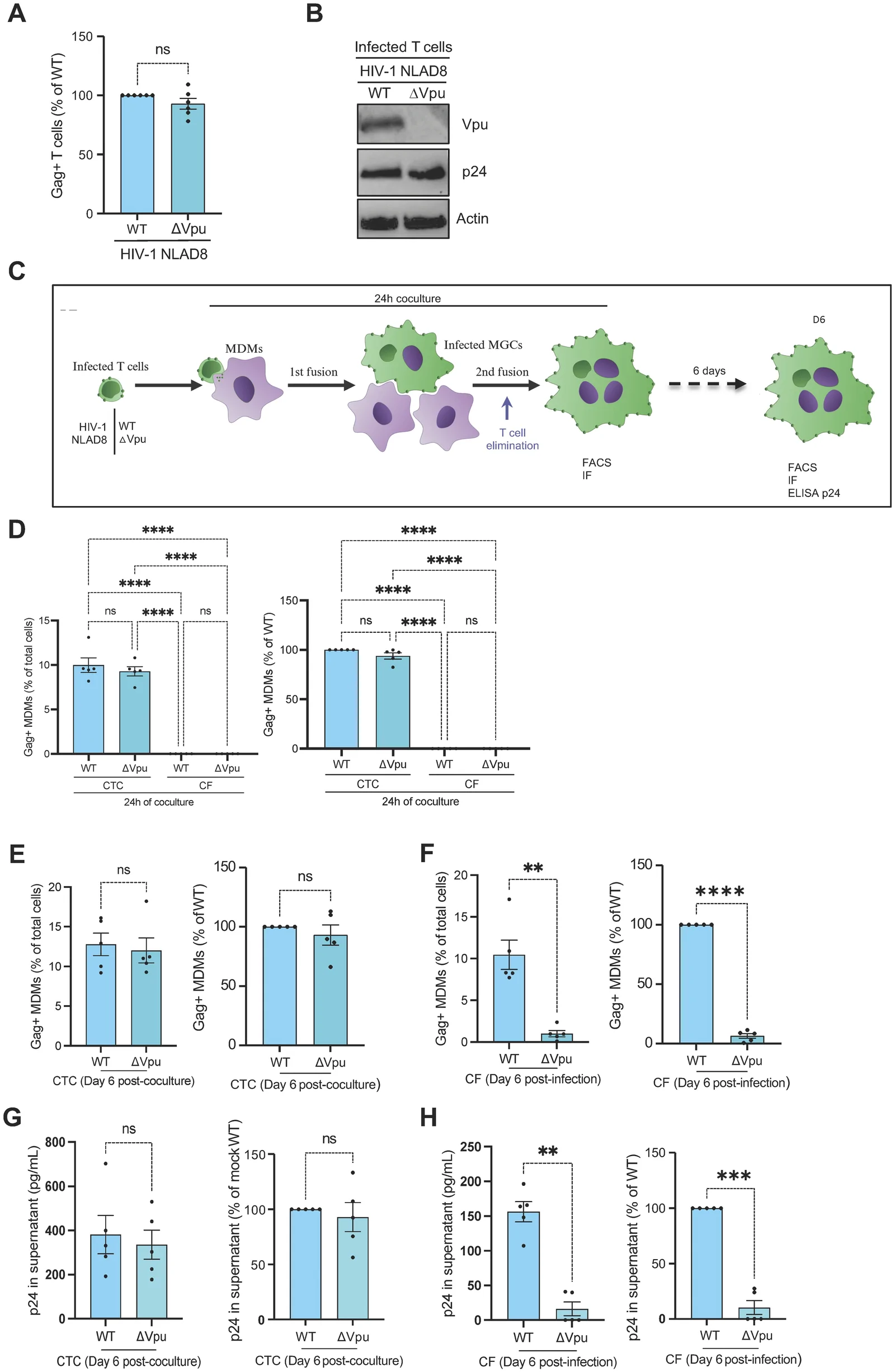
Influence of the Vpu viral protein on HIV-1 cell-to-cell infection of macrophages. **(A and B)** Primary infected CD4 T cells were infected with the WT or ΔVpu HIV-1 NLAD8 strain, and then analyzed 36 h later by flow cytometry after intracellular Gag staining (A) or by Western blotting (B) on cell lysates using antibodies targeting Vpu, Gag (p24) and β-actin. In **(A)**, the results are the means of 6 independent experiments, and are expressed as the percentages of Gag+ T cells relative to the percentage determined with the WT virus (100%). Each dot corresponds to an individual donor. **(C)** Experimental design for analysis of HIV-1 cell-to-cell transfer from infected CD4 T cells to MDMs (created with Biorender). **(D-F)** Primary CD4 T cells were infected with the WT or ΔVpu viruses and cocultured for 24 h with MDMs (CTC) or exposed to cell-free viruses (CF). After elimination of T cells or virus inoculum, MDMs were analyzed immediately (D) or cultured for 6 additional days (E and F) before quantification of the percentages of Gag+ MDMs by flow cytometry. Results are the means of 5 independent experiments performed with CD4 T cells and MDMs from 5 different blood donors. Left panels, results are expressed as the absolute percentage of Gag+ MDMs, while in the right panels, results are expressed as the percentages of Gag+ MDMs determined by virus cell-to-cell or cell-free infection relative to the percentage of MDMs cocultured with WT-infected CD4 T cells (100%). **(G and H)** MDMs were infected by coculture with infected T cells (G) or exposed to cell-free viruses (H) for 24 **h.** After elimination of T cells or cell-free virus inoculum, cell-culture supernatant from MDMs were collected 6 days later, and the viral production was quantified (p24). The results are the means of p24 production quantified from MDM supernatants of 5 independent experiments from 5 different donors. Left panels, results are expressed as the absolute concentrations of p24 in the cell-culture supernatants, while in the right panels, results are expressed as the concentrations of p24 relative to the concentrations measured from the supernatants of the WT-infected MDMs (100%). NI, MDMs cocultured with noninfected CD4 T cells. Error bars represent 1 standard error of the mean (SEM). Statistical significance was determined using the Anova test, and *P* values were obtained by Dunnett’s post-test correction (ns, *P* > 0.05; *, P < 0.05; ****P < 0.0001).

### HIV-mediated cell-cell fusion and MGC formation for cell-to-cell spreading in macrophages are not modulated by Vpu

Since we previously showed that HIV-1 cell-to-cell transfer and dissemination in macrophages were mainly related to cell-cell fusion with the virus-donor infected CD4 T cells [27,28,41], we then evaluated by immunofluorescence (IF) and confocal microscopy whether cell fusion leading to MGC formation was affected when T cells were infected with ΔVpu compared to WT viruses (see Fig 1C for experimental design). As previously, macrophage targets were cocultured for 24 h with WT or ΔVpu infected T cells. Macrophages and MGCs were then stained with anti-Gag antibody for IF analysis, and visualized by confocal microscopy just after coculture or 6 additional days after elimination of T cells. As previously reported [28], Gag+ MDMs cocultured with T cells infected with WT viruses showed a diffuse Gag staining (Fig 2A, and 2E) and were multinucleated when nucleus number per MDM was quantified from the microscopy images (Fig 2B-D and 2F-H). This indicated the formation of infected MGCs after 24 h of coculture or 6 days later after the elimination of T cells, with a mean of nuclei number around 3 nuclei per infected macrophages (Fig 2D and 2H). Interestingly, coculture of MDMs with T cells infected with ΔVpu viruses also led to a similar diffuse Gag staining and the formation of infected MGCs 24 h and 6 days after coculture with ΔVpu infected T cells (Fig 2A-D and Fig 2E-H). Accordingly, all Gag+ macrophages were similarly infected (Infection index, Fig 2B and 2F) with a diffuse Gag staining and contained more than 2 nuclei after 24 h of coculture (Fig 2C and 2D) or 6 days later (Fig 2G and 2H). These results confirm that Vpu expression does not affect cell-to-cell infection of macrophages by cell-cell fusion between infected T cells and macrophage targets for the formation of infected MGCs. Additionally, IF analysis of BST2 localization in non-infected MDMs showed puncta dispersed throughout the cytoplasm and in a perinuclear region (Fig S1A, left images). The same perinuclear punctual staining was observed in cell-to-cell infected MGCs by coculture with WT- or ΔVpu-infected T cells, and no evident co-localization was revealed with the Gag staining (Fig S1A, right panel).

**Fig 2:**
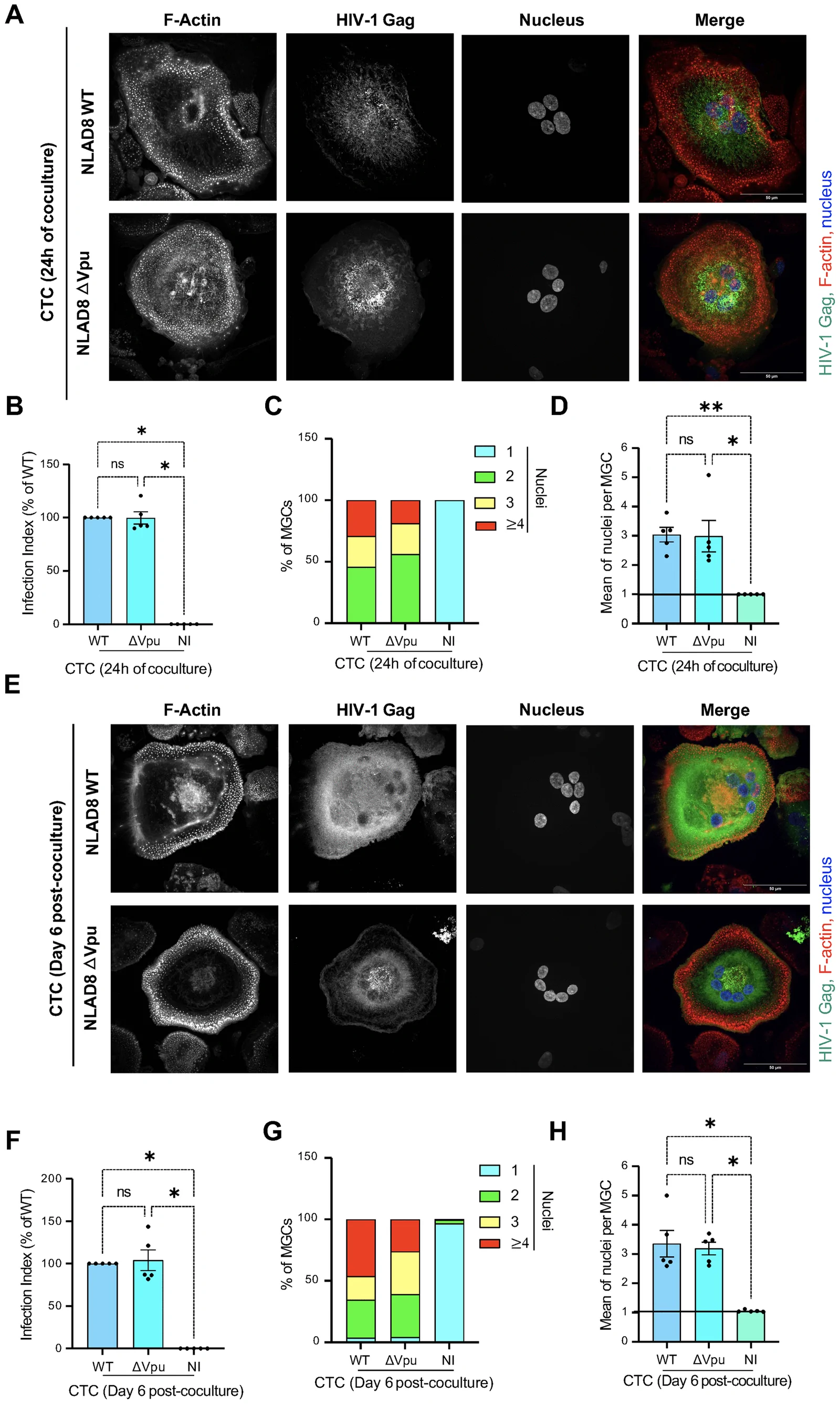
Influence of the Vpu viral protein on HIV-mediated cell-cell fusion of macrophages with infected CD4 T cells. CD4 T cells infected with WT or ΔVpu viruses were cocultured for 24 h with MDMs. After T cell elimination, MDMs were stained immediately after coculture (**A-D**) or 6 days later **(E-H)**, with anti-Gag (green) antibodies, phalloidin (F-actin, red), and the nuclei with Dapi (blue), before observation by confocal microscopy. The total number of nuclei (Dapi+) per Gag+ MDM was quantified on at least 50 cells. In **A** and **E)**, representative images are shown (scale bar, 50 μm). In **B** and **F)**, results are expressed as the level of MDM infection (Infection index). In **C** and **G)**, results are expressed as the percentages of MDMs with 1, 2, 3 or more than 4 nuclei quantified from a representative experiment. In **D** and **H)**, results are expressed as the means of nucleus number per MGC. NI, MDMs cocultured with noninfected CD4 T cells. In **B**, **D**, **F** and **H**, each dot corresponds to an individual donor, and results are the means of 5 independent experiments performed with primary cells from 5 donors. Error bars represent 1 standard error of the mean (SEM). Statistical significance was determined using the Anova test, and *P* values were obtained by Dunnett’s post-test correction (ns, *P* > 0.05; *, P < 0.05; **, P < 0.01).

Together, the results reported in Figs 1 and 2 show that the absence of Vpu in HIV-1 has no impact and is not required for cell-to-cell infection of macrophage targets following fusion with infected T cells.

### HIV-1 cell-to-cell spreading in macrophages by cell-cell fusion escapes type-I interferon treatment independently of expression of the HIV-1 Vpu protein

Most of the host cell restriction factors, including the BST-2/tetherin protein counteracted by Vpu, are stimulated by type I IFN (IFN-I) [42]. As shown in Fig 3A by Western blot analysis, expression of the BST-2 oligomers in non-stimulated macrophages was very low, but greatly enhanced after pretreatment with IFNα(1000 U/mL). Interestingly, BST-2 expression was increased in MDMs infected with either WT or ΔVpu viruses compared to non-infected cells (Fig S1B). We thus investigated the impact of Vpu expression on HIV-1 cell-to-cell infection by cell-cell fusion of macrophages initially stimulated by similar concentration of IFNα. As previously reported [42], the pretreatment of MDMs with 1000 U/mL of IFNα did not significantly impact the rate of cell-to-cell infection of macrophages after 24 h of coculture with T cells infected by WT viruses (Fig 3B, left panel), nor the formation of MGCs after 6 days (right panel). Similarly, when macrophages stimulated by INFα were infected by coculture of Vpu-deleted infected T cells, the percentage of infected macrophages just after coculture or 6 days later was not affected (Fig 3C). In contrast, IFNα treatment totally blocked WT and ΔVpu infection of IFNα-stimulated macrophages infected by cell-free virus particles (Fig 3D). In agreement, virus production in the cell culture supernatant analyzed by p24 ELISA was totally blocked when MDM targets were stimulated by IFNα before infection with cell-free virus particles (Fig 3E, left panel), whereas IFNα had no effect on virus production when IFN-α-stimulated MDMs were infected by coculture with WT- or ΔVpu-infected T cells (Fig 3E, central and right panels).

**Fig 3:**
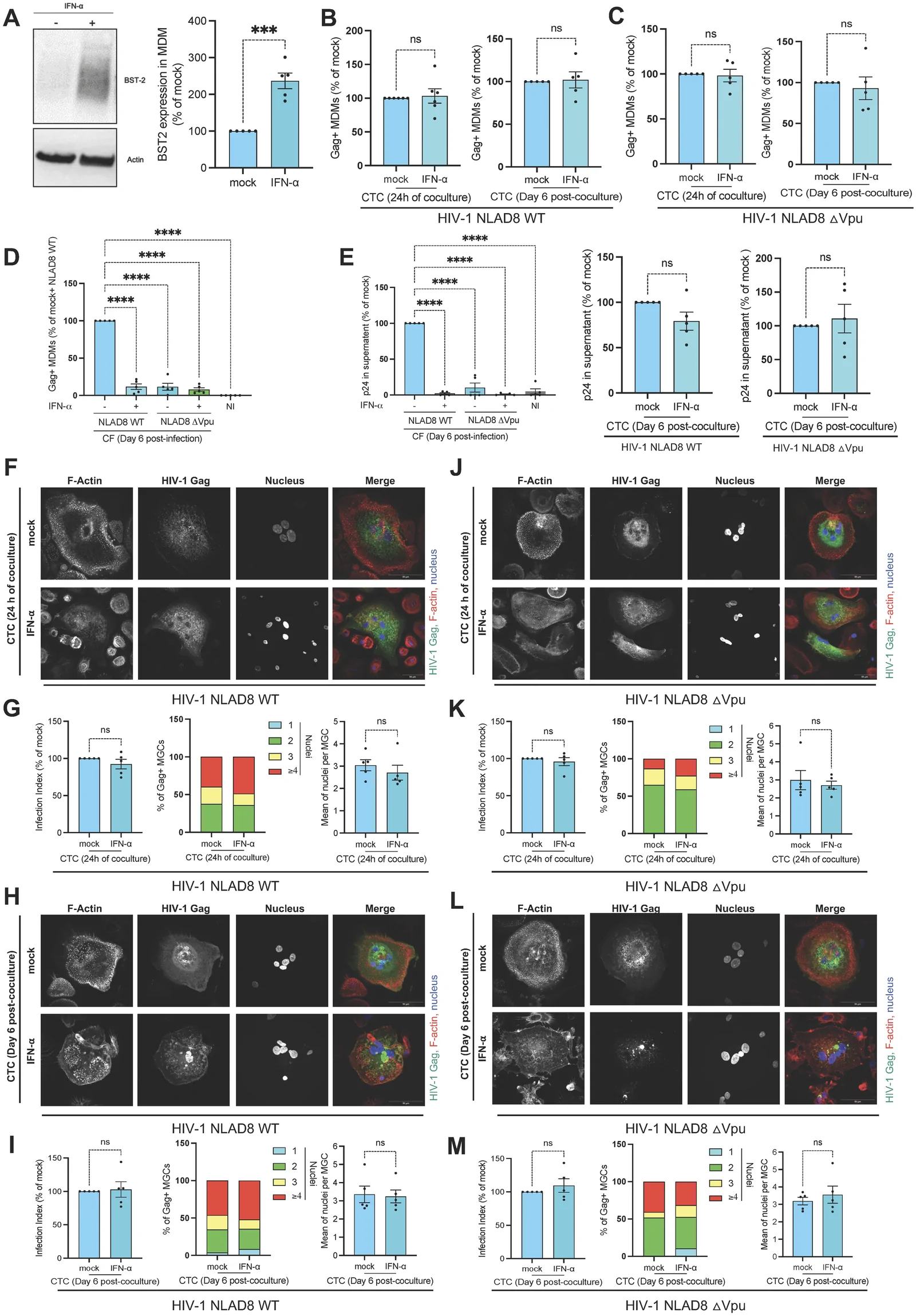
Influence of type-I interferon treatment on HIV-1 cell-to-cell spreading in macrophages by cell-cell fusion. **(A)** MDMs were treated or not (mock) with 1,000 U/mL of IFNα before Western blot analysis using anti-BST-2 and anti-actin antibodies. A representative experiment in shown in the left panel while the right panel corresponds to the quantification of the band intensities from Western blot images; the signal measured in IFNα-treated MDMs was normalized to the signal obtained for actin. Results are expressed as the percentages of the signal intensity measured with IFNα relative to the signal measured without (mock, 100%), and are means of at least 5 independent experiments performed with MDMs of at least 5 different donors. Each dot corresponds to an individual donor. Error bars represent 1 SEM. Statistical significance was determined using the Mann-Whitney U-test (***, *P* < 0.001). **(B and C)** MDMs treated with or without IFNα were cocultured with CD4 T cells infected with WT (B) or ΔVpu (C) viruses for 24 **h.** After elimination of T cells, Gag+ MDMs were analyzed immediately or 6 days later by flow cytometry after Gag staining. Results are expressed as the percentages of Gag+ cells treated with IFNα relative to the percentage of Gag+ measured without (mock, 100%), and are means of 5 independent experiments performed with MDMs of 5 different donors. Statistical significance was determined using the Mann-Whitney U-test (ns, *P* > 0.05). **(D)** MDMs treated with or without IFNα were infected by WT or ΔVpu cell-free viruses and analyzed 6 days later by flow cytometry after Gag staining. Results are expressed as the percentages of Gag+ cells relative to the percentage of Gag+ measured without IFNα treatment and infection with the WT viruses (100%), and are means of 5 independent experiments performed with MDMs of 5 different donors. Statistical significance was determined using the Anova test, and *P* values were obtained by Dunnett’s post-test correction (****, *P* < 0001). **(E)** MDMs were treated with or without IFNα, and then infected by WT or ΔVpu cell-free viruses (left panel) or cocultured for 24 h with T cells infected WT or ΔVpu viruses (central and right panels). After elimination of inoculated viruses or T cells, virus production was quantified by p24 ELISA 6 days later. The results are expressed as the means of p24 production quantified from MDM supernatants of 5 independent experiments from 5 different donors. NI, MDMs cocultured with noninfected CD4 T cells. Statistical significance was determined using the Mann-Whitney U-test (ns, *P* > 0.05). **(F-M)** MDMs treated with or without (mock) IFNα were cocultured with WT (F-I) or ΔVpu (J-M) for 24 h with CD4 T cells before analysis by confocal microscopy just after the 24 h of coculture (F-G and J-K) or 6 days later (H-I and L-M). Representative images are shown in F, H, J and **L)** (scale bars, 50 μm). In G, K, I and M**)**, results are the means of 5 independent experiments performed with primary cells from at least 5 donors. Statistical significance was determined using the Mann-Whitney U-test (ns, *P* > 0.05).

Using IF and confocal microscopy, we next analyzed the impact of IFNα stimulation on the formation of MGCs. As shown in Fig 3F-M, IFNα-stimulated Gag+ MDMs cocultured with T cells infected with either WT (Fig 3F and 3G) or ΔVpu (Fig 3J and 3K) viruses showed similar diffuse Gag staining and were multinucleated (Fig 3G and 3I, central panels), even if MGCs with additional punctate Gag staining were also detected at D6 post-infection (images on Fig 3H and 3L). In addition, IFNα stimulation did not impact the infection index analyzed 24 h after coculture (Fig 3G and 3K, left panels) or 6 days later (Fig 3I and 3M, left panels), or the mean of nuclei in MGCs resulting from cell-cell fusion of WT- or ΔVpu-infected T cells with untreated or IFN-α-treated MDMs (Fig 3G-K and 3I-M, right panels). These results indicate that stimulation by IFNα treatment does not affect cell-cell fusion between infected T cells and macrophage targets for the formation of MGCs, even when MDMs were infected by coculture with T cells infected with ΔVpu viruses.

As it was well established that the late steps of virus assembly and the budding of the virus particles in macrophages take place at the cell surface but also in a virus-containing compartment (VCC) [16,17], we performed transmission electron microscopy analyses for visualization of the ultrastructure of VCCs in MDMs infected by cell-free viruses or virus cell-to-cell transfer in cells treated or not with IFNα. After elimination of the virus inoculum or CD4 T cells, MDMs were examined 6 days later by electron microscopy (Fig 4). As expected, a large VCC containing densely packed mature virus particles was observed in MDMs infected with cell-free WT viruses (Fig 4A, left image). Interestingly, VCCs containing mature viruses were also observed in MDMs infected by virus cell-to-cell transfer, while these latter VCCs seemed to contain less virus particles (Fig 4B and 4C, left images). However, only a few small VCCs containing a few particles were observed in IFNα-stimulated MDMs infected by cell-to-cell transfer initially cocultured with ΔVpu-infected T cells (Fig 4C, lower left image), compared to IFNα-stimulated MDMs cocultured with WT-infected T cells (Fig 4C, upper left image). Of note, virus assembly and budding at the cell surface of MDMs was also observed in all conditions tested (Fig 4, right images). These observations show that VCC-associated assembly and virus budding are largely maintained when MDMs are infected by virus cell-to-cell transfer, but suggest that the absence of Vpu expression can affect organization and ultrastructure of VCCs in IFNα-stimulated MDMs, while not affecting virus release (see Fig 1F and 3E).

**Fig 4:**
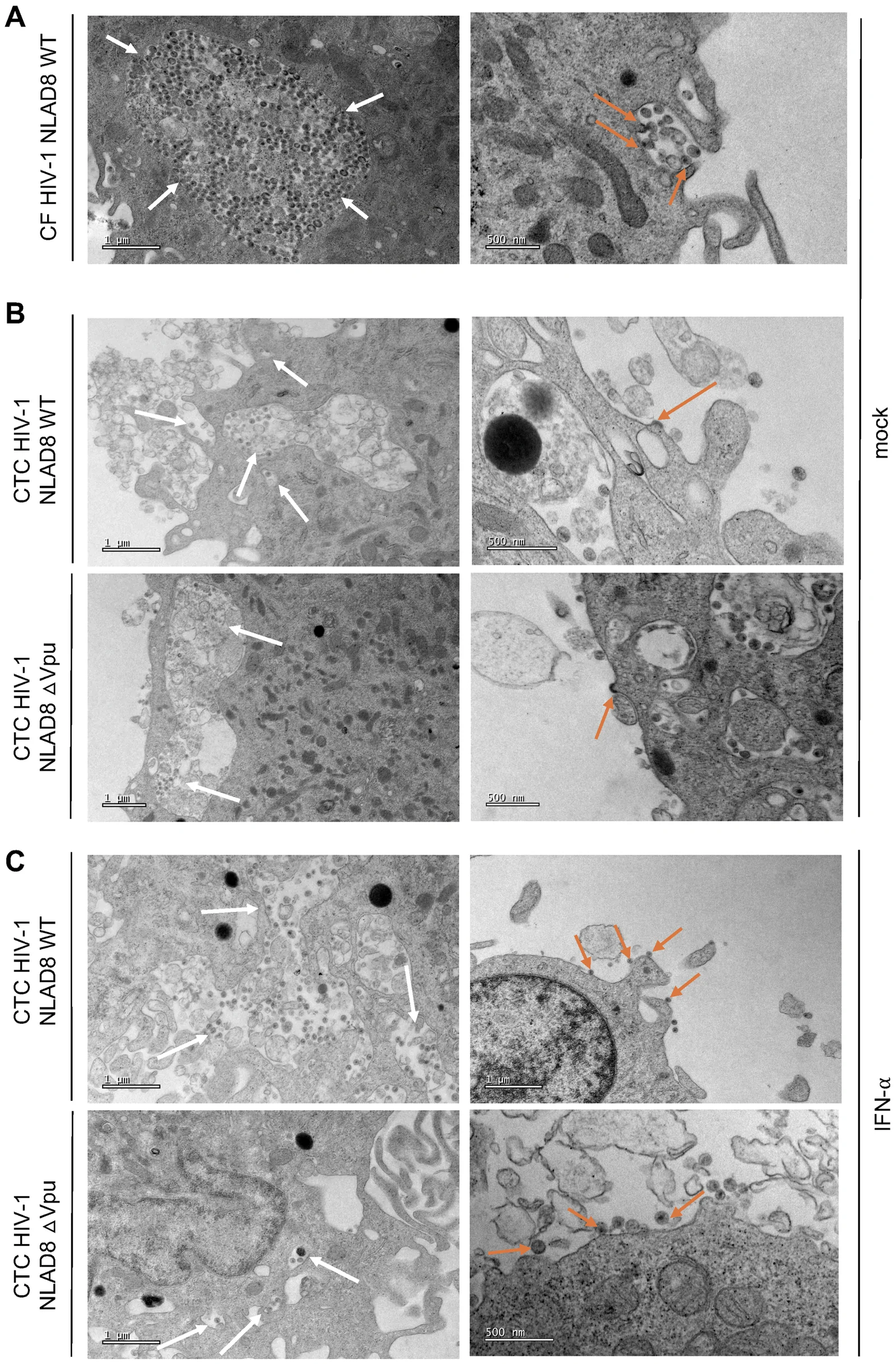
Transmission electron microscopy analysis of macrophage infection. Unstimulated MDMs (**A** and **B**, mock) or MDMs stimulated with IFNα (1000 U/ML) (**C**) were infected with cell-free WT NLAD8 **(A)**, or cocultured with WT- or ΔVpu-infected primary CD4 T cells for 24 h **(B and C)**. After elimination of virus inoculum or infected CD4 T cells, MDMs were cultured for 6 additional days. MDMs were then fixed and dehydrated. Ultrathin sections were cut, stained, and observed with a transmission electron microscope. The images shown are representative of those obtained from analysis of MDMs from 3 independent donors. Left images correspond to the observation of the VCC compartments (white arrows), while the right images correspond to a focus on the cell surface budding virions (orange arrows). Scale bars are indicated.

### Depletion of IFN-I-stimulated BST-2 in infected T cells or MDMs does not impact on HIV-1 cell-to-cell infection of macrophages

Among the various functions of the HIV-1 Vpu, one of the main function of this auxiliary protein is to counteract the BST-2/tetherin protein, which is the best-characterized and most potent IFN-stimulated restriction factor acting on the late steps of the viral life cycle [13]. Therefore, we investigated the direct role of BST-2 for cell-to-cell infection of IFNα-stimulated macrophages. Differentiated MDMs were transfected with siRNA targeting BST-2 before pretreatment with IFNα, and then cocultured with infected CD4+ T cells. As evidenced in Fig 5A, Western blot analysis showed that the IFNα-induced upregulation of BST-2 was largely reduced by siBST-2 treatment, whereas expression of BST-2 was still detected in macrophages treated with the control siRNA (siCtrl). Of note, the constitutive expression of APOBEC3G (Fig 5A, left panel) was not affected in MDMs transfected with either siCtrl or siBST-2 in this experiment even when cells were stimulated with IFNα [28]. BST-2-depleted MDMs were then pretreated with IFNα (1000 U/ml) and cocultured with autologous primary CD4 T cells infected with WT or ΔVpu viruses. As shown in Fig 5B and 5C, no differences in virus cell-to-cell infection of IFNα-stimulated MDMs were observed in BST-2-depleted MDMs infected with the WT or ΔVpu viruses when analyzed by flow cytometry (panels B and C, respectively), immediately after coculture or 6 days later. Similar results were observed when IFNα-stimulated MDMs were cocultured with T cells infected with a mutated virus (A15LW23A) that specifically fails to counteract BST2 [43] (Fig S2). In agreement, no significative modifications of virus production measured by p24 ELISA 6 days after the initial coculture were observed in IFNα-stimulated MDMs infected by coculture with either WT or ΔVpu viruses (Fig 5D, left and right panels, respectively). Similarly, no differences in the formation of infected MGCs analyzed by confocal microscopy after Gag staining were observed with IFNα-stimulated MDMs transfected with siBST-2 or siCtrl, and quantified either 24 h after coculture or 6 days later with WT- or ΔVpu-infected T cells (Fig 5E-H and 5I-L, respectively).

**Fig 5:**
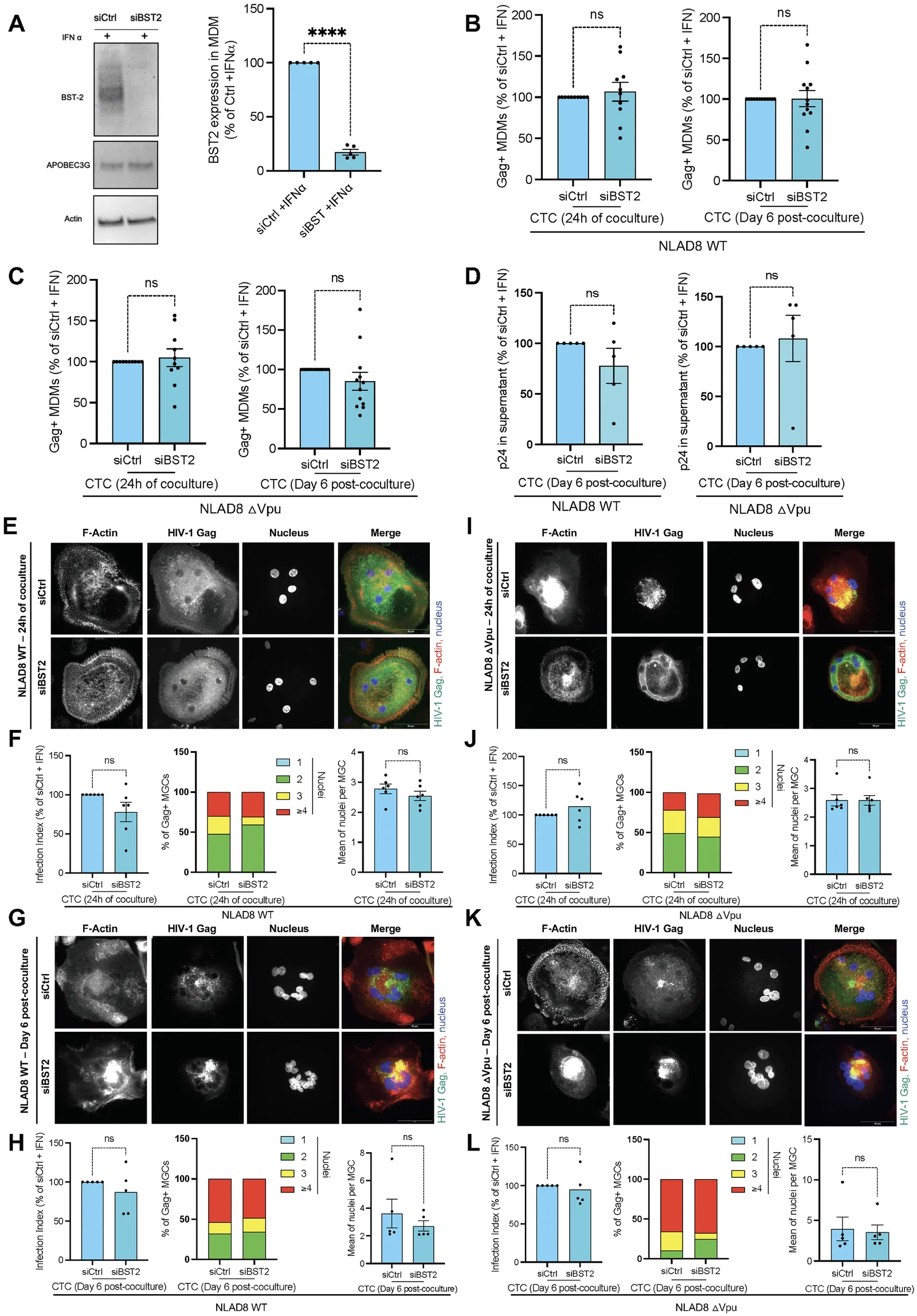
Impact of depletion of IFN-I-stimulated BST-2 on HIV-1 cell-to-cell infection of macrophages. **(A)** MDMs transfected with siBST-2 or siCtrl were treated with 1,000 U/mL of IFNα before Western blot analysis using anti-BST-2, APOBEC3G and actin antibodies. A representative experiment in shown in the right panel while the left panel corresponds to the quantification of the BST-2 band intensities from Western blot images, and the signal measured in siRNA-treated MDMs were normalized to the signal obtained for actin. Results are expressed as the percentages of the signal intensity measured in siBST-2 transfected MDMs relative to the signal measured with siCtrl, and are means of 5 independent experiments performed with MDMs of 5 different donors. Each dot corresponds to an individual donor. Error bars represent 1 SEM. Statistical significance was determined using the Mann-Whitney U-test (****, *P* < 0.0001). (**B** and **C**) siRNA-transfected MDMs were treated with IFNα before coculture for 24 h with CD4 T cells infected with WT (B) or ΔVpu (C) viruses. After elimination of T cells, Gag+ MDMs were analyzed immediately or 6 days later by flow cytometry after Gag staining. Results are expressed as the percentages of Gag+ MDMs transfected with siBST-2 relative to the percentages of Gag+ MDMs transfected with siCtrl (100%), and are means of at least 8 independent experiments performed with MDMs of at least 8 different donors. Statistical significance was determined using the Mann-Whitney U-test (ns, *P* > 0.05). **(D)** MDMs transfected with siBST-2 or siCtrl were treated with IFNα, and were then cocultured with WT (left panel) or ΔVpu (right panel) infected CD4 T cells. Virus production was analyzed 6 days later by p24 ELISA. Results are expressed as the percentages of p24 measured in cell-culture supernatant of siBST-2 transfected MDMs relative to p24 measured in supernatant of siCtrl transfected cells (100%), and are means of 5 independent experiments performed with MDMs of 5 different donors. Statistical significance was determined using the Mann-Whitney U-test (ns, *P* > 0.05). **(E-L)** MDMs transfected with siBST-2 or siCtrl were pretreated with IFNα and cocultured for 24 h with CD4 T cells infected with either WT (E-H) or ΔVpu (I-L) viruses before analysis by confocal microscopy just after the 24 h of coculture (E-F and I-J) or 6 days later (G-H and K-L). Representative images are shown in E, G, I, and K (scale bars, 50 μm). In F, J, H and **L)**, results are the means of at least 5 experiments performed with primary cells from at least 5 donors. Statistical significance was determined using the Mann-Whitney U-test (ns, *P* > 0.05).

Finally, we investigated the impact of BST-2 depletion in infected virus-donor T cells on virus cell-to-cell transfer to MDMs. BST-2 expression was detected in primary CD4 T cells and was stimulated by IFNα treatment (S3A Fig), while infection had no significant impact on BST-2 expression (S3B Fig). Infected T cells were thus transfected with siBST-2 before pretreatment with IFNα(S3C Fig), and then cocultured with infected MDMs. As shown in S3D and S3E Fig, depletion of BST-2 in infected T cells had no significant impact on cell-to-cell transfer and spreading in MDMs of WT and ΔVpu viruses. Together, the results reported in Fig 5 and S3 show that cell-to-cell infection of macrophages and formation of MGCs is not restricted by the IFNα-stimulated BST-2 protein expressed in virus-donor T cells or MDM target cells, even when virus-donor T cells are infected with ΔVpu viruses.

### HIV-1 cell-to-cell infection of macrophages is still sensitive to the nelfinavir protease antiviral inhibitor

We previously showed that HIV-1 cell-to-cell infection of macrophages was largely resistant to antiretroviral drugs acting on the early steps of the viral life cycle, including nucleoside and non-nucleoside reverse-transcriptase inhibitors (zidovudine and nevirapine), integrase inhibitors (raltegravir) and the PF74 capsid-targeting compounds [28]. We thus investigated here whether this cell-to-cell mode of macrophage infection might also impact on the susceptibility to protease inhibitors, such as nelfinavir (NFV), acting on the late step of virus replication. MDMs were pretreated with 1 or 10 μM of NFV, and 10 μM of AZT used as a control of antiretroviral drug acting on the early step of virus replication. After coculture with infected T cells, MDM infection was first analyzed by flow cytometry 24 h after coculture or 6 days later (Fig 6A, left and central panels). As expected, AZT and NFV had only modest effects when MDM infection was analyzed just after 24 h of coculture (left panel). As we previously reported [28], AZT had no significative inhibitory effect on cell-to-cell macrophage infection analyzed 6 days later, whereas NFV showed a significant dose-dependent inhibitory effect on cell-to-cell infection of macrophages evidenced at the concentrations of 1 and 10 μM (Fig 6A, central panel). By contrast, both AZT and NFV showed a significant strong inhibitory activity at 10 μM when macrophages were analyzed 6 days after infection with cell-free viruses (Fig 6A, right panel). Similarly, when analyzed by immunofluorescence and confocal microscopy, MGCs were formed after the 24 h of coculture even in the presence of AZT and NFV antiretroviral drugs (Fig 6B and 6C). However, NFV significantly inhibited MGC formation in a dose-dependent manner at 6 days post-coculture, whereas AZT had no antiviral activity at the same 10 μM concentration (Fig 6D). Accordingly, viral production analyzed 6 days after coculture was resistant to AZT when MDMs were infected by cell-to-cell virus transfer but was totally inhibited when MDMs were pretreated with NFV even at 1 μM (Fig 6E). As expected, viral production by MDMs was abolished by both AZT and NFV when infected by cell-free virus particles (Fig 6F). These results confirm that viral cell-to-cell infection of MDMs are resistant to drugs as AZT acting on the early steps of the viral life cycle as shown previously, whereas HIV-1 cell-to-cell macrophage infection remains sensitive to the protease antiviral NFV inhibitor acting on the late steps of virus replication.

**Fig 6:**
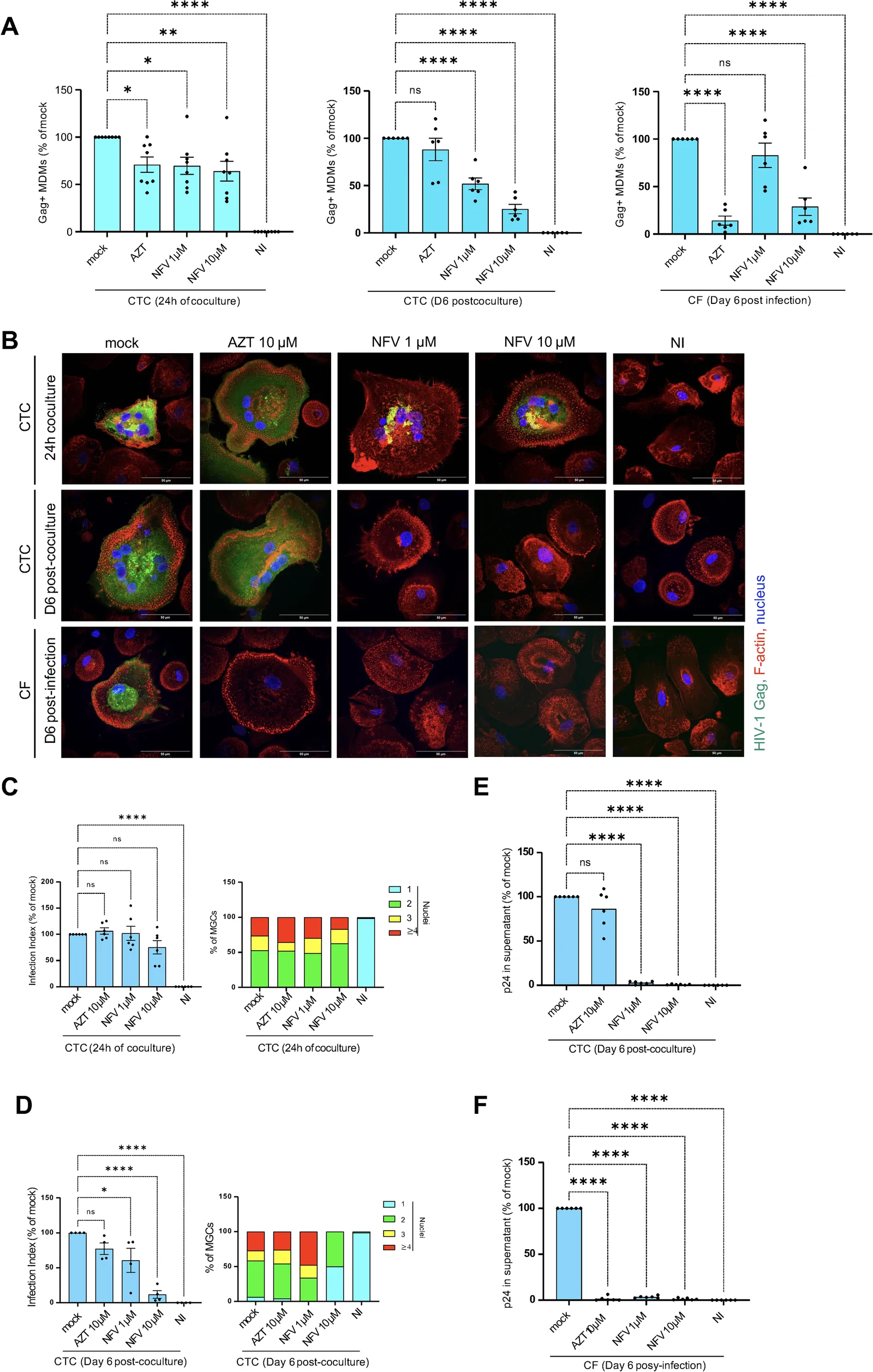
Susceptibility of HIV-1 cell-to-cell infection of macrophages to the nelfinavir protease antiviral inhibitor. **(A)** MDMs pretreated with AZT (10 μM) or Nelfinavir (1 and 10 μM) were infected with cell-free viruses (right panel) or cocultured for 24 h with infected CD4 T cells and analyzed just after coculture (left panel) or 6 days later (middle and right panels) by flow cytometry after Gag staining. Results are the means of at least 6 independent experiments performed with MDMs and CD4 T cells from at least 6 different donors, and are expressed as the percentages of Gag+ MDMs relative to that determined without drugs (mock, 100%). Each dot corresponds to an individual donor. Error bars represent 1 SEM. Statistical significance was determined using the Anova test, and *P* values were obtained by Dunnett’s post-test correction (ns, *P* > 0.05; *, *P* < 0.05; **, *P* < 0.01; ****, *P* < 0.0001). **(B-D)** MDMs pretreated with AZT, NFV, or without drug (mock), were inoculated with cell-free viruses (CF) or cocultured (CTC) with CD4 T cells for 24 **h.** After elimination of virus inoculum or T cells, MDMs were analyzed by confocal fluorescence microcopy just after the coculture, or cultured for 6 additional days. The total number of nuclei per Gag+ MDM was quantified on at least 50 cells. Representative images are shown in **(B)** (scale bars, 50 μm). NI, MDMs cocultured with noninfected CD4 T cells. In **C** and **D)**, results were analyzed just after the 24 h coculture (**C**) or 6 days later **(D)**, and are expressed as infection index (left panels) and represent the means of at least 4 independent experiments performed with CD4 T cells and MDMs of 4 different donors, or as the percentage of MGCs with 1, 2, 3, or more than 4 nuclei quantified from a representative experiment (right panels). **(E and F)** MDMs pretreated or not with AZT or NFV were cocultured with infected CD4 T cells (**E**) for 24 h or infected by cell-free viruses **(F)**. After elimination of the virus inoculum or T cells, virus production was analyzed 6 days later from MDM supernatants of 6 independent experiments performed with MDMs of 6 different donors. Results are expressed as the percentages of p24 production relative to that determined without drugs (mock, 100%). Statistical significance was determined using the Anova test, and *P* values were obtained by Dunnett’s post-test correction (ns, *P* > 0.05; *, *P* < 0.05; ****, *P* < 0.0001).

## Discussion

We previously demonstrated that the main mechanism of HIV-1 cell-to-cell infection was independent of expression of the viral auxiliary proteins mainly involved in counteraction of host cell factors acting on the early steps of virus replication, such as SAMHD1, APOBEC3 and SERINC proteins [25,28]. We now report that this route of macrophage infection also escapes the restriction by BST-2 acting on the last steps of virus replication. In agreement, the absence of BST-2 restriction is independent of the viral Vpu protein mainly involved in counteraction of this cellular factor. Together, these observations highlight how the virus cell-to-cell infection of macrophages upon cell-cell fusion with infected T cells is so efficient for HIV-1 spreading as compared to infection of macrophages by cell-free virus particles [11,44–48].

In addition, we previously showed that the virus-mediated cell-cell fusion was also largely resistant to antiviral drugs acting on the early steps of the virus life cycle, such as reverse-transcriptase, capsid and integrase inhibitors [28]. Our results reported here show that HIV-1 cell-to-cell infection of macrophages is still sensitive to the protease antiviral inhibitors, such as Nelfinavir, acting by inhibition of virus particle maturation during the very late step of the viral life cycle.

When infected by cell-free viruses, most host cell restriction factors of HIV-1, such as SAMHD1 and members of the SERINC, IFITMs, TRIM and APOBEC3 proteins, inhibit entry or post entry early steps of virus replication, before integration of the viral DNA into the host genome [42]. In agreement, stimulation of MDMs by IFNα totally inhibited cell-free virus replication and spreading [28]. By contrast, we previously showed that IFNα stimulation of SAMHD1 and APOBEC3G and 3A expression did not affect cell-to-cell infection of macrophages [25,28].

Interestingly, the BST-2/tetherin protein is the best characterized restriction factor stimulated by type I IFN that acts in the late steps of the virus life cycle by tethering *de novo* produced virus particles at the cell surface of infected cells [13]. This restriction activity results in a limitation of virus budding and release from the cell surface of the target cells, including macrophages [43,49,50]. Compared to cell-free infection, we confirm that IFN stimulation of macrophages leading to high BST-2 expression has no impact on virus spreading by cell-to-cell fusion, independently of the viral Vpu counteracting protein [28]. This is contrasting to many studies showing that cell-to-cell spreading between CD4 T cell through the formation of the virological synapse was still restricted by BST-2 [50–52]. A single study showed that cell-to-cell spread of HIV-1 overcame the BST-2-mediated restriction in T cells [53]. We can hypothesize that the restriction activity of BST-2 is overcome by the high production of viral components, including Gag, related to the presence of several copies of integrated DNA in all MGC nuclei when MDMs are infected by virus cell-to-cell transfer [28]. In addition, we cannot exclude that differential expression of Env at the cell surface of T cells infected with WT or ΔVpu viruses may also influence the efficiency of virus cell-to-cell transfer from infected T cells to macrophages. Intriguingly, our findings could also indicate that all other IFN-stimulated genes, such as IFITM, MX2, Viperin, and MARCH proteins [42], fail to affect HIV-1 cell-to-cell infection of macrophages by initial cell-cell fusion with infected T cells, as we already reported for APOBEC3 protein family, including APOBEC3G and 3A [28].

In macrophages infected by cell-free viruses, the assembly of the viral components and the budding of new infectious virus particles can specifically take place in a specialized intracellular membrane compartment called VCC, for virus-containing compartment [16,17]. This VCC was described as a protected compartment, distinct from endosomes or multivesicular bodies, in continuity with the plasma membrane and connected to the extracellular space by thin channels. The interplay between the cell surface function of BST-2 on virus budding and the formation of VCCs in infected macrophages is still being discussed [10,43]. Since our electron microscopy observations showed that the formation of VCCs was largely maintained when MDMs were infected by virus cell-to-cell transfer, it will be interesting to analyze the formation of VCCs in BST-2-depleted MDMs infected by cell-free or cell-associated viruses.

The auxiliary Vpu protein is specifically expressed by HIV-1 for counteraction of the tethering activity of IFN-induced BST-2, thus allowing efficient release of virus particles from the cell surface of infected cells [13]. Vpu counteracts the BST-2 restriction activity by sorting of BST-2 to lysosomal compartments for degradation [54–56]. In contrast, our data demonstrated that the BST-2-induced restriction of virus budding was bypassed and/or overcome when macrophages were infected by cell-cell fusion with infected virus-donor infected T cells. We can hypothesize that the absence of the BST-2 restriction activity observed for virus spreading by cell-cell fusion could be related to a different mechanism of assembly and budding of mature newly formed virus particles from the VCCs. Further investigations are thus needed for analysis of the formation of VCCs when macrophages are infected by cell-free viruses or by virus cell-to-cell transfer [43]. These investigations should help to decipher how the cell-cell fusion route of macrophage infection is able to bypass and/or overcome the BST-2 restriction activity on virus release. Of note, a single study regarding the role of Vpu on virus cell-to-cell infection of macrophages reported opposite results indicating that Vpu could enhance the susceptibility of macrophage to infection [26]. However, this effect was related to the limited process of phagocytosis of dying apoptotic infected T cells [23,27], whereas infection by virus-mediated cell-cell fusion is the main route of macrophage infection of living non-apoptotic infected T cells [27] and could explain the differences of these results with our present observations.

Finally, it is surprising that cell-to-cell infection of macrophages by cell-cell fusion is still sensitive to drugs such as Nelfinavir that specifically inhibit virus infectivity through targeting of the viral protease, whereas we recently showed that this mode of virus cell-to-cell spreading is resistant to all the antiviral drugs acting in the pre-integration steps of the viral life cycle [28]. However, this susceptibility to protease inhibitors of the cell-cell fusion mode of macrophage infection could be related to the fact that these antiprotease drugs are the only antiretroviral compounds acting after virus budding and release of virus particles. In contrast, intracellular early steps of the virus life cycle are targeted and inhibited by all other antiretroviral drugs, such as reverse transcriptase and integrase inhibitors, as well as the PF74 inhibitor targeting the viral capsid for inhibition of nuclear translocation of the viral DNA. These differences in the mode of action between drugs acting on preintegration intracellular steps and antiprotease could explain that these latter inhibitors are still active to block virus cell-to-cell infection of macrophages. Further investigations are needed to understand why and how HIV-1 cell-to-cell spreading in macrophages is affected by drugs acting on virus maturation.

In summary, this study further explores the mechanisms underlying the very efficient cell-cell fusion mechanism for HIV-1 cell-to-cell spreading in macrophage target cells. This mode of virus dissemination is significantly different and original in terms of mechanisms for cellular and molecular viral replication affecting the effects of host cell restriction factors, especially those stimulated by IFN [57,58]. While HIV-1 infection of macrophages by cell-free viruses is strongly restricted by IFN-stimulated genes [28,42], this cell-to-cell mechanism of virus spreading in macrophages by cell-cell fusion escapes the restriction imposed by host cell factors, including SAMHD1, SERINC, APOBEC3 and BST-2 proteins. Importantly, it leads to the formation of highly virus-productive MGCs reminiscent of the infected MGCs originating from macrophages observed *in vivo* in infected patients and SIV-infected macaques [29–39]. Therefore, characterizing the cellular and molecular mechanisms of cell-to-cell viral spreading in macrophages leads to a better understanding of: i) transmission of cell-associated HIV-1; ii) virus spreading between HIV-1 target cells in tissues, and iii) susceptibility to antiretroviral drugs. Importantly, the cell-to-cell mode of infection of macrophages can surely influence the establishment of long-lived viral macrophage reservoirs in tissues [2–6,8,9,59,60], the main obstacle to HIV-1 eradication in patients living with HIV [1]. These mechanisms of virus spreading are likely important *in vivo* since shared by many other viruses, including HIV-1, but also Herpesviridae, Paramyxoviridae and Coronaviridae, leading to cell-cell fusion between infected cells and neighboring cells as evidenced in tissues, such as brain, lungs and lymphoid and non-lymphoid tissues, of infected patients [2,5,21,61,62].

## Materials and methods

### Plasmids and reagents

The proviral pNLAD8 was obtained from the AIDS Research and Reference Reagent Program, NIAID, while the WT, ΔVpu, and A15LW23A HIV-1 proviral plasmids (HIV-1 ADA strain) were obtained from Eric Cohen (Université de Montréal, Canada). The pVSVg plasmid encoding the VSV-G envelope glycoprotein has been described [24,25,41]. The pNLAD8 plasmid deleted of the HIV-1 Vpu auxiliary proteins (ΔVpu) was constructed from the wild-type pNLAD8 plasmid as described [28]. Briefly, the pNLAD8ΔVpu was constructed through insertion of the *EcoR*I-*BamH*I fragment of the pNL4.3Vpu- plasmid (obtained from the AIDS Research and Reference Reagent Program) into the pNLAD8 previously digested by *EcoR*I and *BamH*I. The following antibodies were used: RD1- or fluorescein isothiocyanate (FITC)-conjugated anti-Gag (clone KC57, Beckman Coulter); Alexa Fluor 647- or Fluor 555-conjugated phalloidin (Life Technologies); anti-BST-2 antibody was purchased from Proteintech; anti-Vpu anti-APOBEC3G (anti-Apo17) and anti-Env (G12) antibodies were obtained from the AIDS Research and Reference Reagent Program; anti-p24 antibody was obtained from Abcam; and anti-β-actin was purchased from Invitrogen [25]. The antiretroviral compounds AZT and NFV were obtained from the AIDS Research and Reference Reagent Program, while IFNα2b was purchased from Miltenyi Biotech.

### Cell culture

HEK293T and Jurkat cell lines were obtained from the ATCC, and were maintained in culture as described [41]. Blood samples from anonymous healthy donors were purchased from “*Etablissement Français du Sang*” (EFS, the French National Blood Agency, Rungis, France). Monocytes were purified from blood of healthy donors using a CD14-positive selection kit (CD14 microbeads; Miltenyi) according to manufacturer’s guidelines. Monocytes were then differentiated into macrophages for 8 days in culture medium supplemented with 25 ng/ml of macrophage colony-stimulating factor (M-CSF) (Miltenyi) as described [27,41]. Human primary CD4+ T cells were isolated from blood of healthy donors using a CD4-positive selection kit (CD4 microbeads; Miltenyi) according to manufacturer’s guidelines, and maintained in culture as described [27,41].

### Virus production and titration

Wild-type and ΔVpu HIV-1 NLAD8 strains, as well as WT, ΔVpu and A15LW23A ADA strains, were produced in HEK293T cells as previously described [28,41]. The amounts of viral p24 produced were determined by enzyme-linked immunosorbent assay (ELISA; Innogenetics) as recommended by the manufacturer. Viral titers were determined using primary CD4 T cells and flow cytometry as described previously [63]. For production of viruses dedicated to the cell-free infection experiments, viral stocks were used at a multiplicity of infection (MOI) of 0.5 to infect T cells for 24 h. After washing, cells were cultured for another 24 h, and the amount of Gag p24 produced in the cell-culture supernatant was determined by ELISA as described [41].

### Cell-free and cell-to-cell viral transfer and dissemination in MDMs

For cell-free infection of MDMs, viruses were used at a concentration of 250 ng/mL of viral p24 for 10^6^ MDMs as described [27]. After 24 h of infection and elimination of the viral inoculum, the level of Gag+ MDMs was analyzed by flow cytometry as previously described [41], either just after the 24 h of infection or after culture for 6 additional days. For cell-to-cell virus infection assays of MDMs from infected primary purified CD4 T cell, cells were carried out as described previously [24,25]. Briefly, primary CD4 T cells were infected with virus stocks produced from HEK293T cells at a MOI of 0.5 for 24 h, and then for another 48 h after an intermediate washing step. Where indicated, MDMs were pretreated before coculture for 1 h with antiretroviral drugs (AZT and NFV) at the concentration of 1 μM or 10 μM, or overnight with IFNα2b at the indicated concentration (1,000 U/mL), and then cocultured at a 2:1 cell ratio, without or with either antiretroviral drugs or IFNα2b, with infected T cells for 24 h as described [41]. When used, AZT and NFV were maintained during the coculture and in the culture medium after removal of T cells. T cells were eliminated by washes in PBS and 2 mM EDTA-containing PBS [24]. The percentages of Gag+ MDMs were analyzed by flow cytometry using the KC57 anti-Gag mAb (1/500) just after the coculture or after 6 additional days in culture [24]. Cell-culture supernatants were also harvested for quantification of the virus production by p24 ELISA as described [24,25]. Similarly, MDMs depleted of BST-2 after transfection of specific siRNA (Dharmacon) as described [25,28] were cocultured with infected primary CD4 T cells, depleted or not of BST-2, and then analyzed by flow cytometry or for viral production in the cell-culture supernatant as described above [25,28].

### Fluorescence microscopy

To visualize virus cell-to-cell transfer by cell-cell fusion, infected primary CD4 T cells were cocultured for 24 h with MDMs plated onto coverslips as described [24,41]. Where indicated, MDMs were pretreated before coculture with IFNα2b (1,000 U/mL) or antiretroviral drugs (AZT and NFV at the concentration of 1 μM or 10 μM) as described above [24,41]. After elimination of T cells, MDMs were directly analyzed after the coculture or cultured for 6 additional days, before fixation, blocking and staining with Dapi as described [41]. MDMs were then permeabilized and stained using KC57 FITC-conjugated anti-Gag antibody (1/200 dilution), and phalloidin-Alexa Fluor 647 (Molecular Probes), diluted in permeabilization buffer for 1 h. Coverslips were then washed with PBS and mounted on slides using 10 μl of Fluoromount (Sigma). Images were acquired on a spinning-disk confocal system (IXplore; Olympus) based on an inverted motorized fluorescence microscope (IX83; Olympus) equipped with a Yokogawa CSU-W1 spinning-disk head, a 10-position filter wheel, and a camera port, and were then processed using Fiji software (ImageJ; NIH). Quantitative image analysis and determination of Dapi+ nuclei were analyzed from images of at least 50 cells followed by processing using Fiji as described [24,25].

### Transmission electron microscopy

Primary CD4 T cells infected with the WT or ΔVpu HIV-1 NLAD8 strain as described above [24,25], were cocultured for 24 h with MDMs that had been plated onto coverslips with or without IFNα (1000 U/mL). In parallel, MDMs were infected by cell-free NLAD8 for 24 h. After elimination of CD4 T cells or virus inoculum, MDMs were cultured for 6 additional days. The coverslips were then fixed using 2.8% glutaraldehyde and 2% PFA for 2 h. After 3 washes in PBS, the cells were dehydrated and embedded into epoxy (Electron Microscopy Sciences) as described previously [24,25]. Ultrathin sections of 90 nm were cut with an ultramicrotome, stained with uranyl acetate and Reynold’s lead, and observed with a transmission electron microscope (JEOL 1011). Acquisition was performed with a Gatan ES1000W charge-coupled-device camera.

### Western blotting

For analysis of expression of Vpu, p24 and BST-2 in infected T cells, and APOBEC3G and BST-2 in MDMs, cells (10^6^) were lysed in 100 μl of reducing Laemmli sample buffer (2x) supplemented with PhosSTOP (phosphatase inhibitor cocktail tablets; Sigma-Aldrich) and cOmplete (EDTA-free protease inhibitor cocktail; Sigma-Aldrich), and then boiled at 96°C for 10 min. Cell lysates were then resolved by SDS-PAGE as described [25,28], and analyzed by Western blotting using anti-Vpu (1/1000), anti-HIV-1 p24 (1/1000), anti-APOBEC3G (1/1000), anti-BST-2 (1/1000), and anti-β-actin (1/1000) antibodies.

### Statistical analysis

Statistics and curve fitting were performed using the GraphPad prism software. As mentioned in the figure legends, when comparisons were performed between two groups, two-tailed t-tests were used for statistical analyses. When comparisons were performed between more than 2 groups, ANOVA tests were used for statistical analyses, and *P* values were obtained by Dunnett’s post-tests correction to compare every mean to the control group mean. For all our experiments, graphics represent the results of at least 5 independent experiments performed with primary cells of at least 5 different donors (n = 5 donors). Since each experiment was conducted using primary MDMs from different donors, the results for each experiment were normalized to the control group.

## Supporting information

S1 Fig BST-2 localization and expression in virus cell-to-cell infected MDMs. CD4 T cells infected with WT or ΔVpu viruses were cocultured for 24 h with MDMs. After T cell elimination, MDMs were analyzed by immunofluorescence 6 days later (A) and by Western blot just after the 24h-coculture (B). In A), cells were stained with anti-Gag (green) and anti-BST-2 antibodies (light blue), phalloidin (F-actin, red), and the nuclei with Dapi (dark blue), before observation by confocal microscopy. Representative images are shown (left images, scale bars, 50 μm), and the right panel corresponds to the quantification of BST-2/Gag colocalization evaluated by calculating Mander’s coefficient on at least 20 cells using ImageJ. Each dot represents 1 individual cell. Statistical significance was determined using the Mann-Whitney U-test (ns, *P* > 0.05). In B), Lysates from infected cells were analyzed by Western blotting using anti-BST-2 and anti-actin antibodies. The band intensities were quantified using NIH Image software, and the signal measured in infected cells was normalized to the signal obtained for actin. The indicated values are expressed as percentages of the signal intensity relative to NI (100%) and are representative of 2 independent experiments.
(TIFF)

S2 Fig Cell-to-cell transfer of mutated Vpu viruses in macrophages. Primary CD4 T cells were infected with the WT, Vpu-deleted (ΔVpu) or Vpu-mutated A15LW23A (AW) viruses and cocultured for 24 h with IFNα-stimulated MDMs. After elimination of T cells, MDMs were analyzed immediately (A) or cultured for 6 additional days (B) before quantification of the percentages of Gag+ MDMs by flow cytometry. Results are the means of 3 independent experiments performed with CD4 T cells and MDMs from 3 different blood donors. Results are expressed as the percentages of Gag+ MDMs determined by cell-to-cell infection relative to the percentage of MDMs cocultured with WT-infected CD4 T cells (100%). Each dot corresponds to an independent blood donor. Statistical significance was determined using the Anova test, and *P* values were obtained by Dunnett’s post-test correction (ns, *P* > 0.05).
(TIFF)

S3 Fig Impact of depletion of BST-2 in virus-donor infected T cells on HIV-1 cell-to-cell infection of macrophages. (A) CD4 T cells were treated or not for 48 h with 1,000 U/mL of IFNα before Western blot analysis using anti-BST-2, and actin antibodies. (B) T cells were infected with WT or ΔVpu viruses for 48 h before Western blot analysis. (C) T cells were transfected with siCtrl or siBST-2 and then infected with WT viruses for 48 h before Western blot analysis. In the left panels, representative experiments are shown, while the right panels correspond to the quantification of the BST-2 band intensities from Western blot images, and the BST-2 signal was normalized to the signal obtained for actin. Results are expressed as the percentages of the BST-2 signal intensity measured in T cells relative to the signal measured in control infected T cells, and are means of at least 3 independent experiments performed with T cells of at least 3 different donors. Each dot corresponds to an individual donor. In A and C), Statistical significance was determined using the Mann-Whitney U-test (ns, P > 0.05; *, P < 0.05; **, P < 0.01), while in B), statistical significance was determined using the Anova test, and the P value was obtained by Dunnett’s post-test correction (ns, P > 0.05). (D and E) siRNA-transfected CD4 T cells were infected with WT (D) or ΔVpu (E) viruses before coculture for 24 h with MDMs. After elimination of T cells, Gag+ MDMs were analyzed immediately (left panels) or 6 days later (right panels) by flow cytometry after Gag staining. Results are expressed as the percentages of Gag+ MDMs transfected with siBST-2 relative to the percentages of Gag+ MDMs transfected with siCtrl (100%), and are means of 4 independent experiments performed with T cells and MDMs of 4 different donors. Statistical significance was determined using the Mann-Whitney U-test (ns, P > 0.05).
(TIFF)

S1 Raw Images Raw images in this study. (PDF)

## Fundings

This work was supported in part by CNRS, Inserm and the University Paris Cité (SB). This project has received financial support from the CNRS through the MITI interdisciplinary programs (SB and AD). It was also funded by grants from the “*Agence Nationale de Recherche sur le SIDA et les Hépatites Virales (ANRS-MIE)*” (SB). SY was supported by grants from the China Scholarship Council (CSC). MW was supported by grants from *ANRS-MIE* and “*Fondation pour la Recherche Médicale*” *(FRM)*. AD was supported by a grant from the French National Centre for Scientific Research (CNRS, MITI interdisciplinary programs). The funders had no role in study design, data collection and analysis, decision to publish, or preparation of the manuscript.
