# Supplementary material for: HIV-1 cell-to-cell infection of macrophages escapes restriction by the BST-2 host cell factor independently of the Vpu counteracting viral protein
Source: PLoS Pathog. 2026 Oct 23;22(10):e1014702. doi: 10.1371/journal.ppat.1014702 (PMC13743038; doi:10.1371/journal.ppat.1014702)
Supplement: Supplementary file 1 [file ppat.1014702.s004.pdf]

Original uncropped and unadjusted image corresponding to Figure 1B

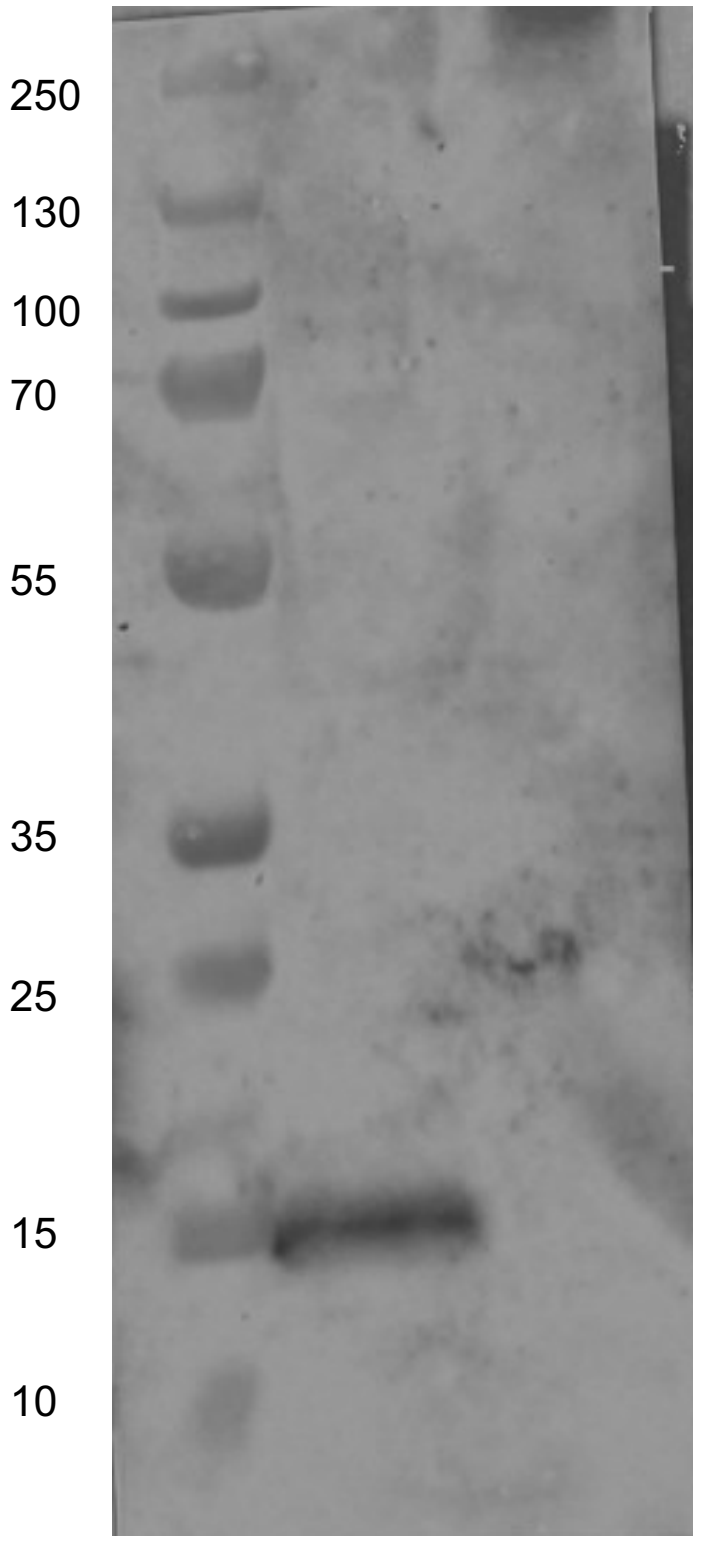

Detected protein: Vpu
Membrane: PVDF
Detection method: ECL (ChemiDoc MP)
Exposure time: 15s

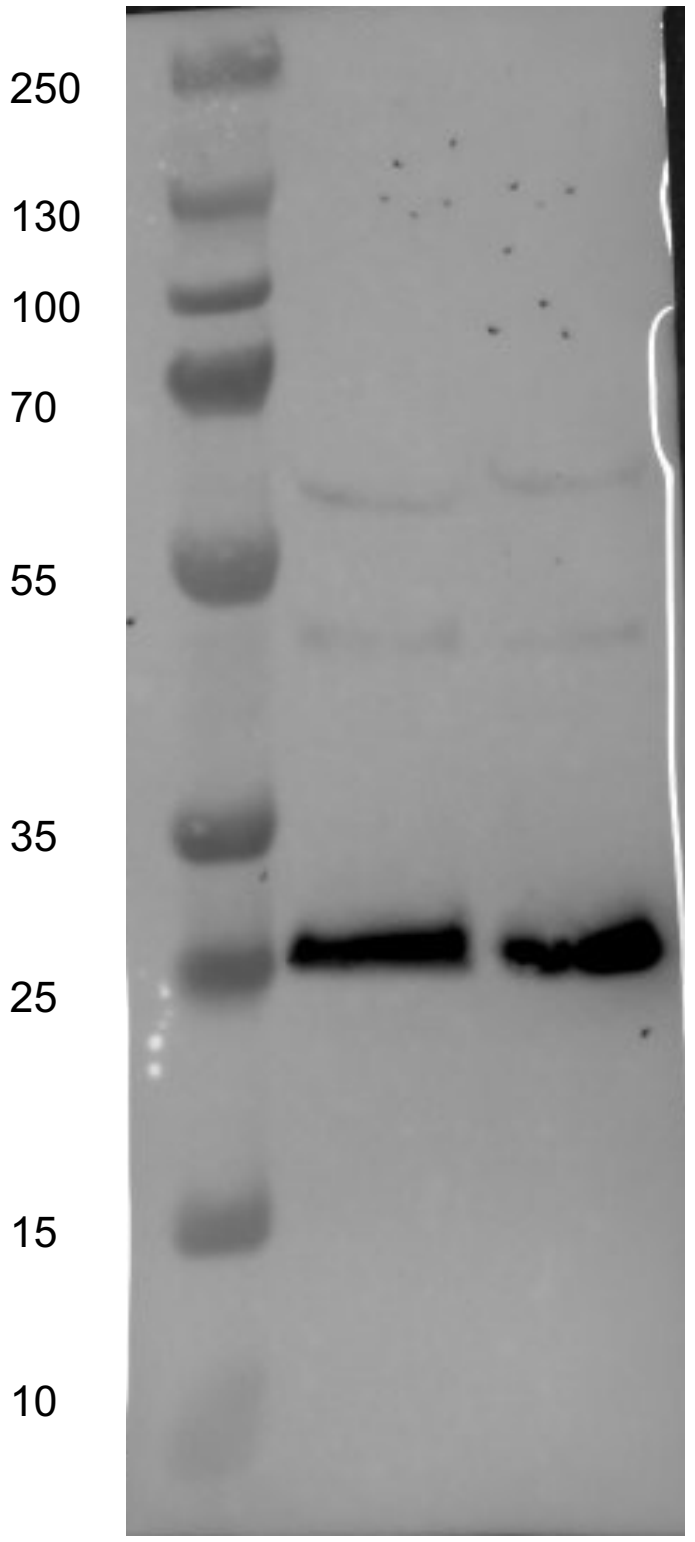

Detected protein: p24
Membrane: PVDF
Detection method: ECL (ChemiDoc MP)
Exposure time: 10s

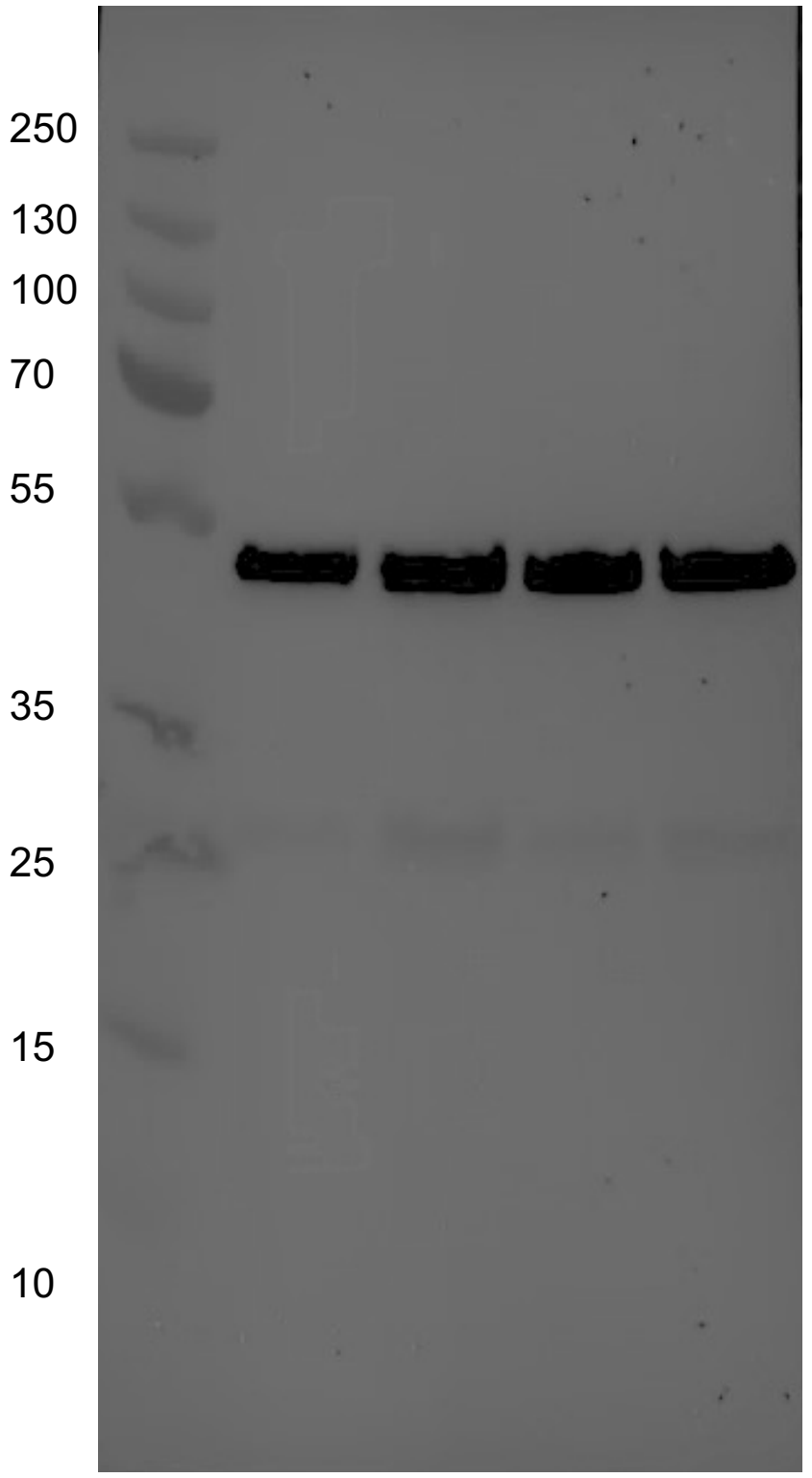

Detected protein: Actin
Membrane: PVDF
Detection method: ECL (ChemiDoc MP)
Exposure time: 2s

X=lane not included in final figure

Original uncropped and unadjusted image corresponding to Figure 3A

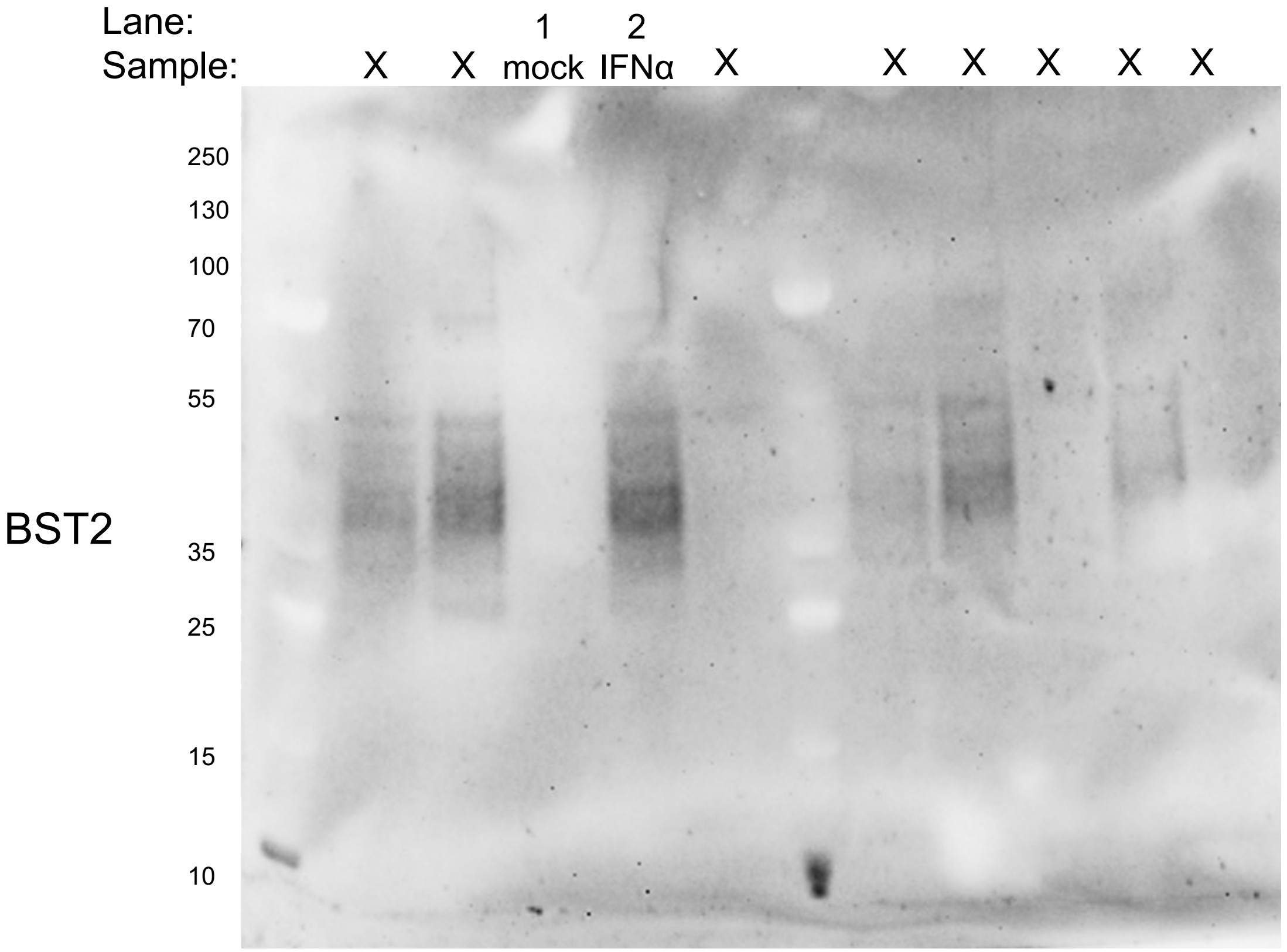

Detected protein: BST2
Membrane: PVDF
Detection method: ECL (ChemiDoc MP)
Exposure time: 150s

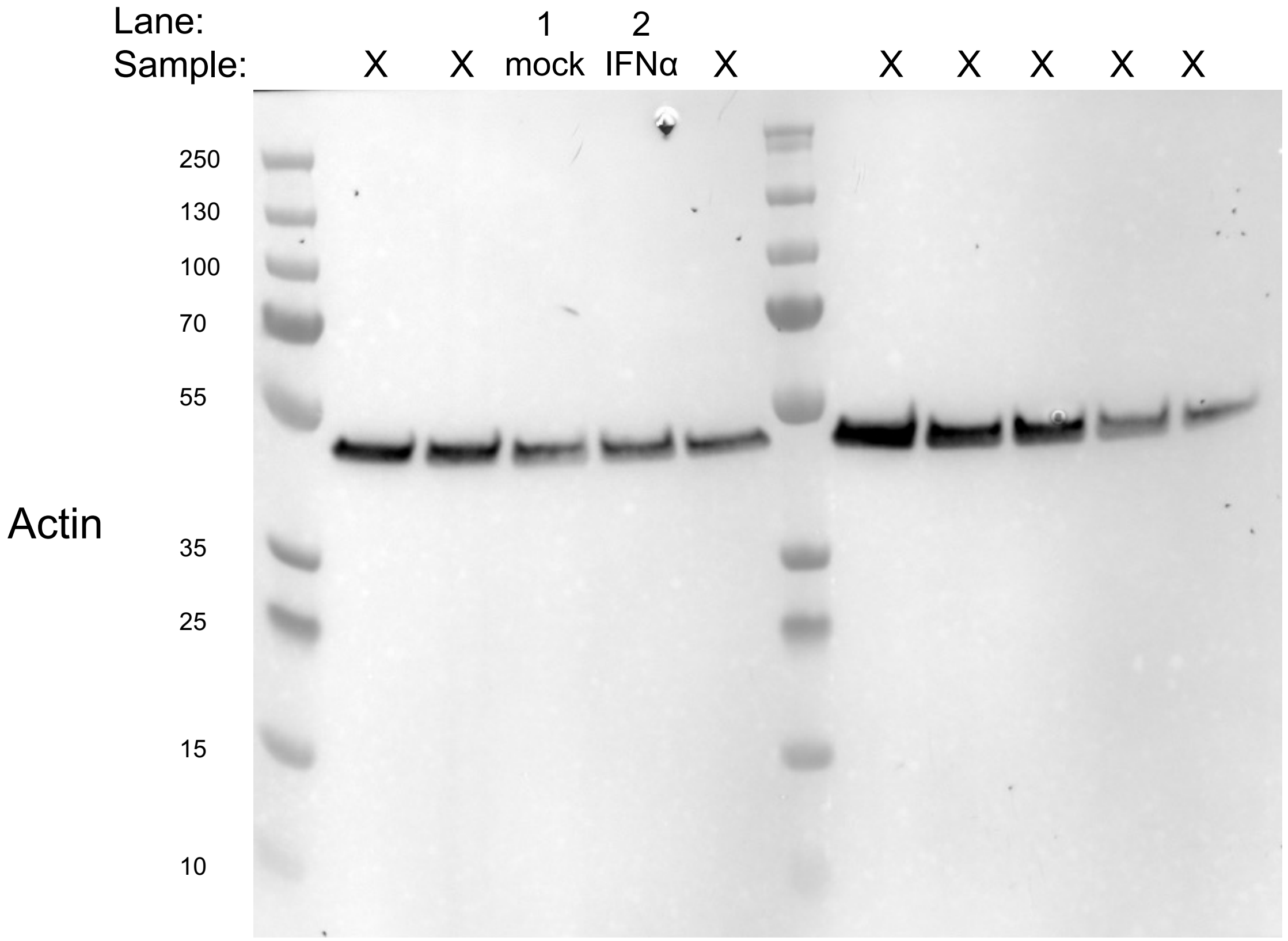

Detected protein: Actin
Membrane: PVDF
Detection method: ECL (ChemiDoc MP)
Exposure time: 3s

X=lane not included in final figure

Original uncropped and unadjusted image corresponding to Figure 5A

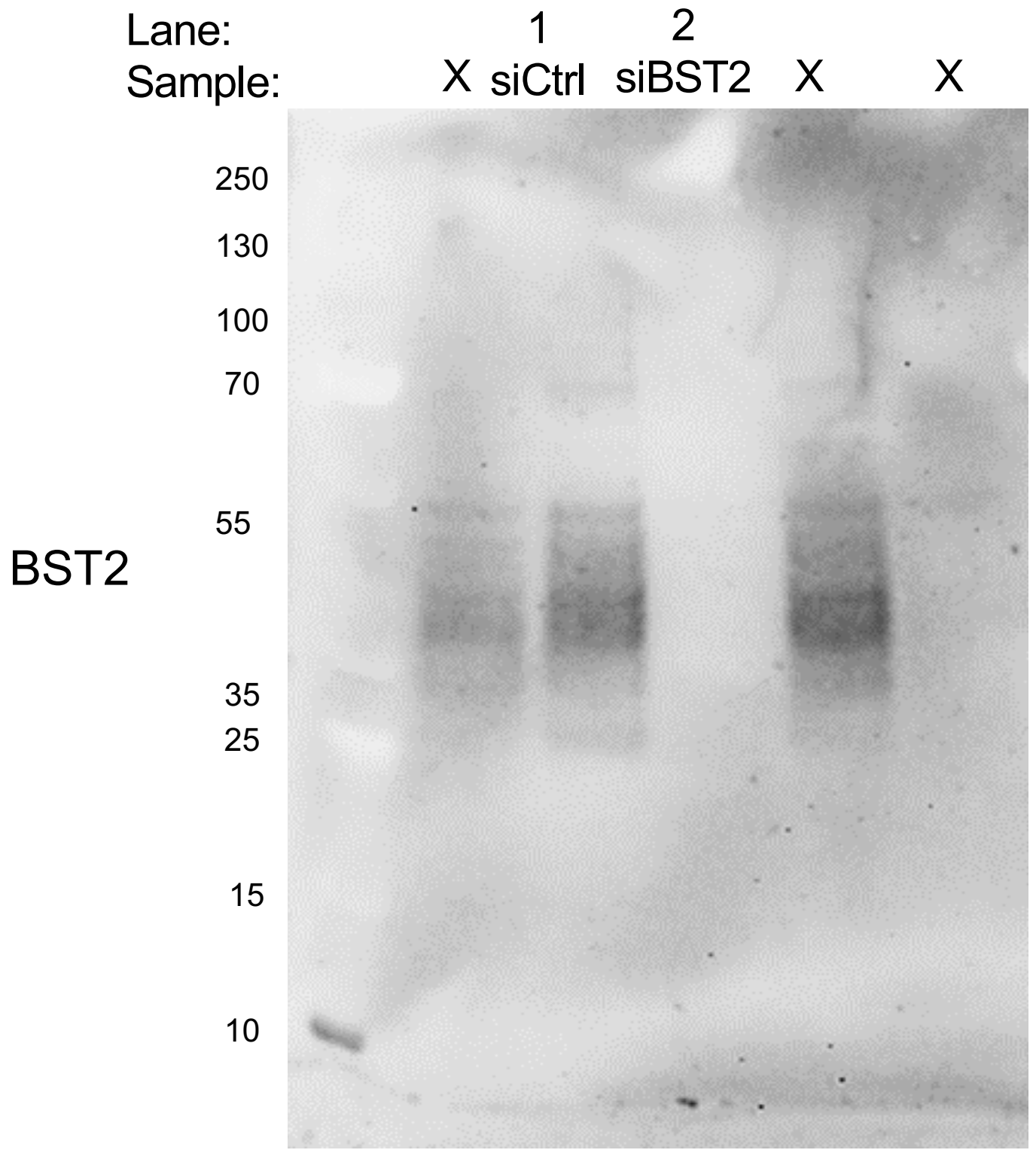

Detected protein: BST2
Membrane: PVDF
Detection method: ECL (ChemiDoc MP)
Exposure time: 180s

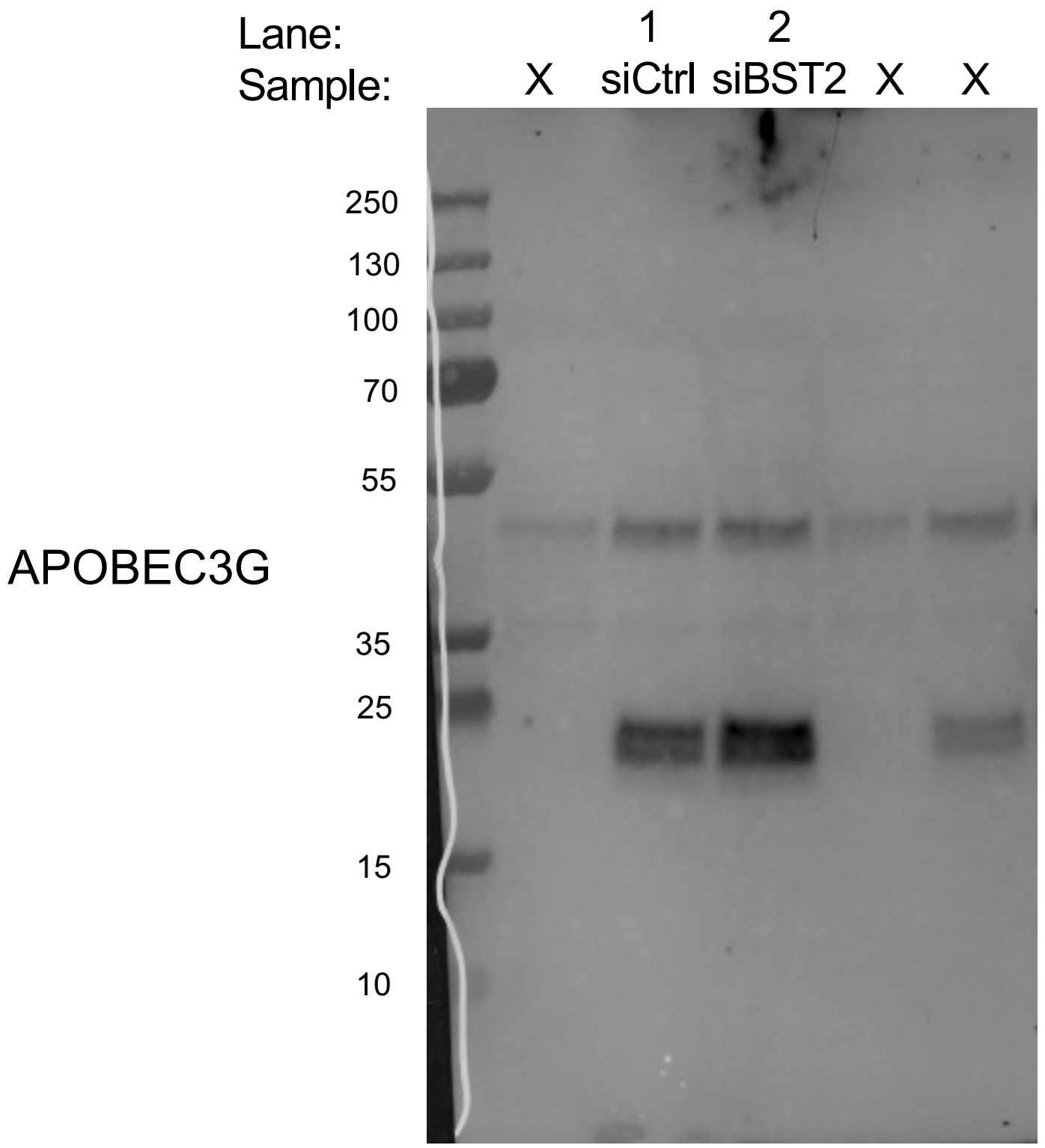

Detected protein: APOBEC3G
Membrane: PVDF
Detection method: ECL (ChemiDoc MP)
Exposure time: 90s

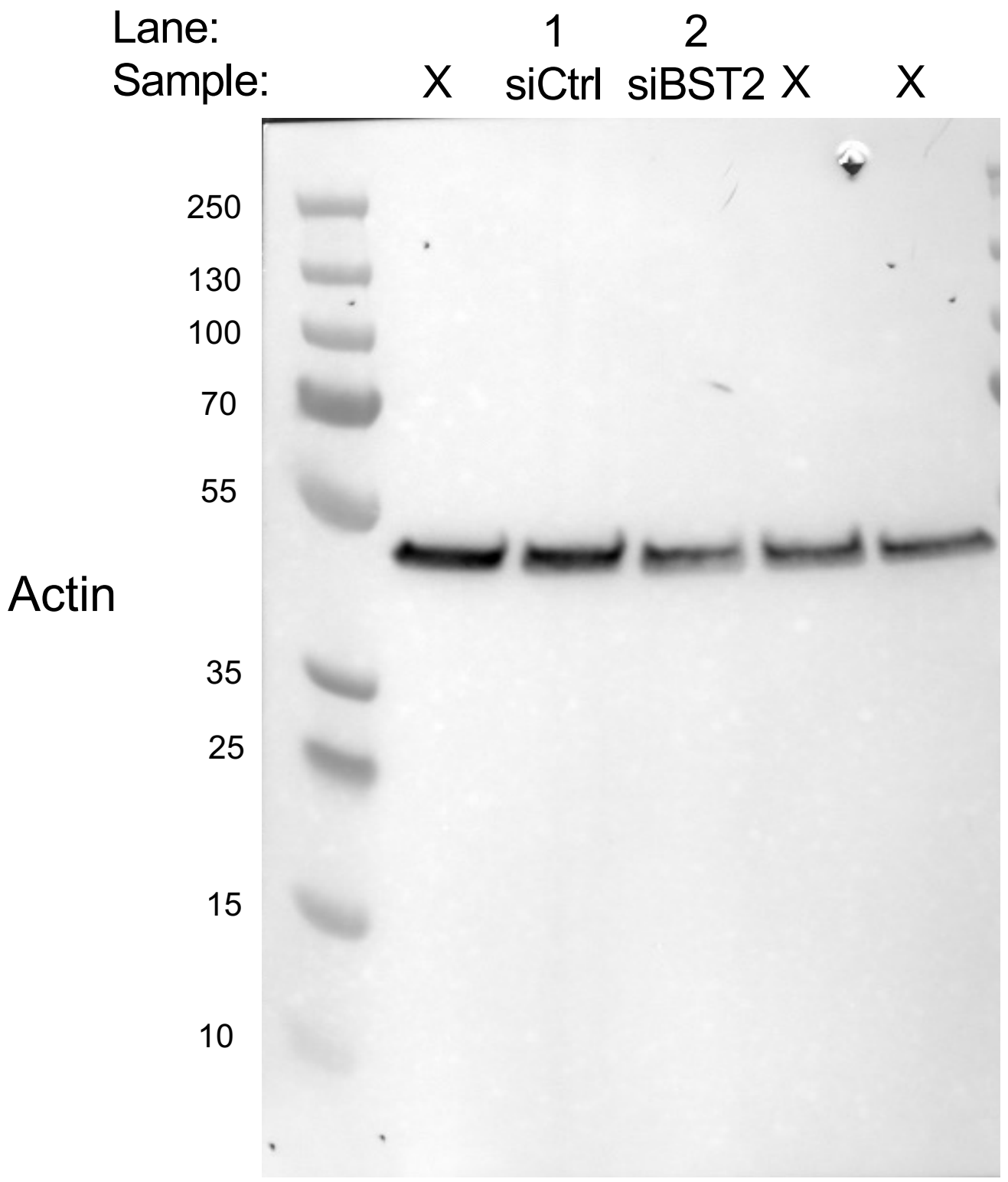

Detected protein: Actin
Membrane: PVDF
Detection method: ECL (ChemiDoc MP)
Exposure time: 4s

X=lane not included in final figure

Original uncropped and unadjusted image corresponding to Figure S2B

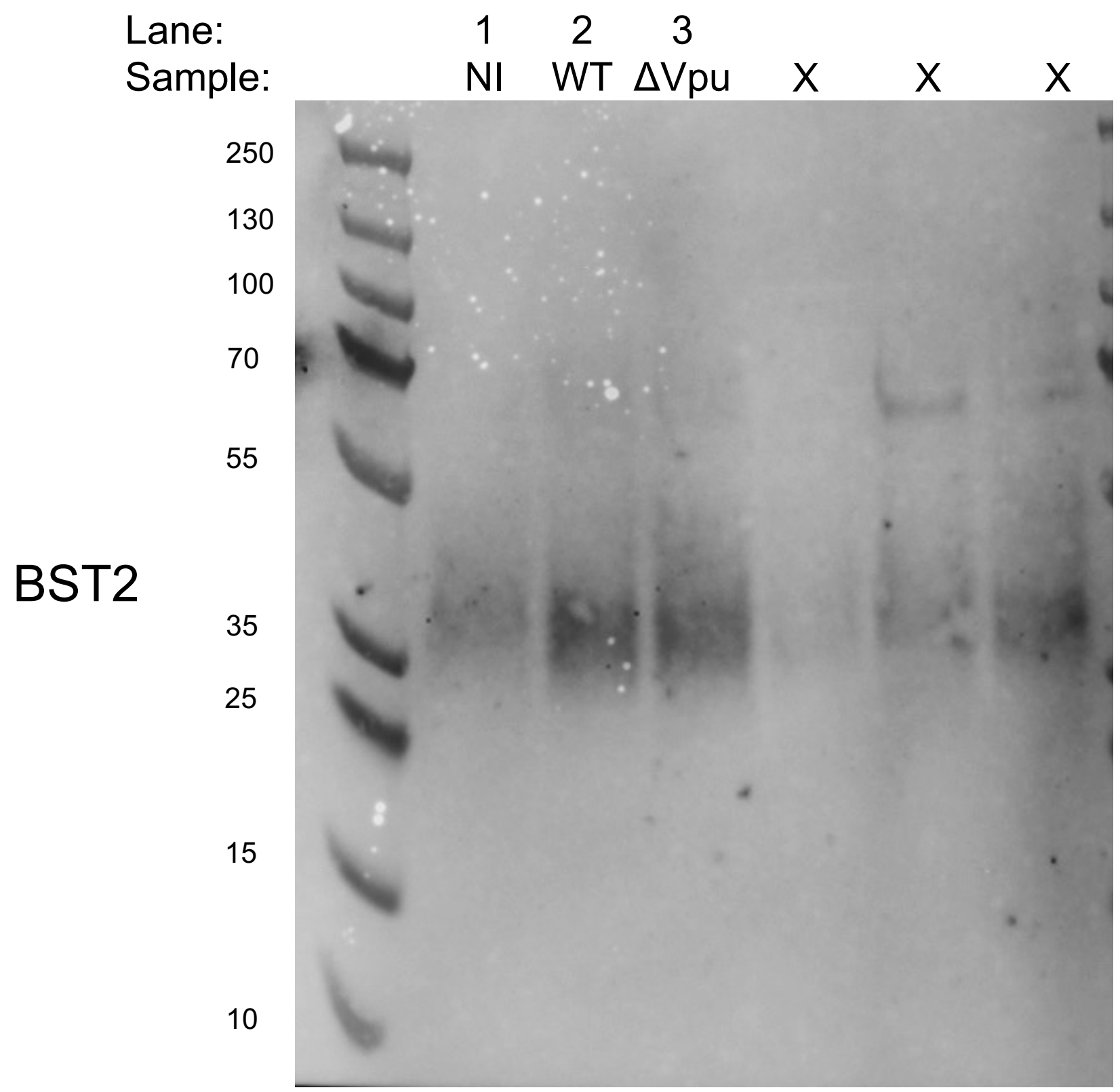

Detected protein: BST2
Membrane: PVDF
Detection method: ECL (ChemiDoc MP)
Exposure time: 180s

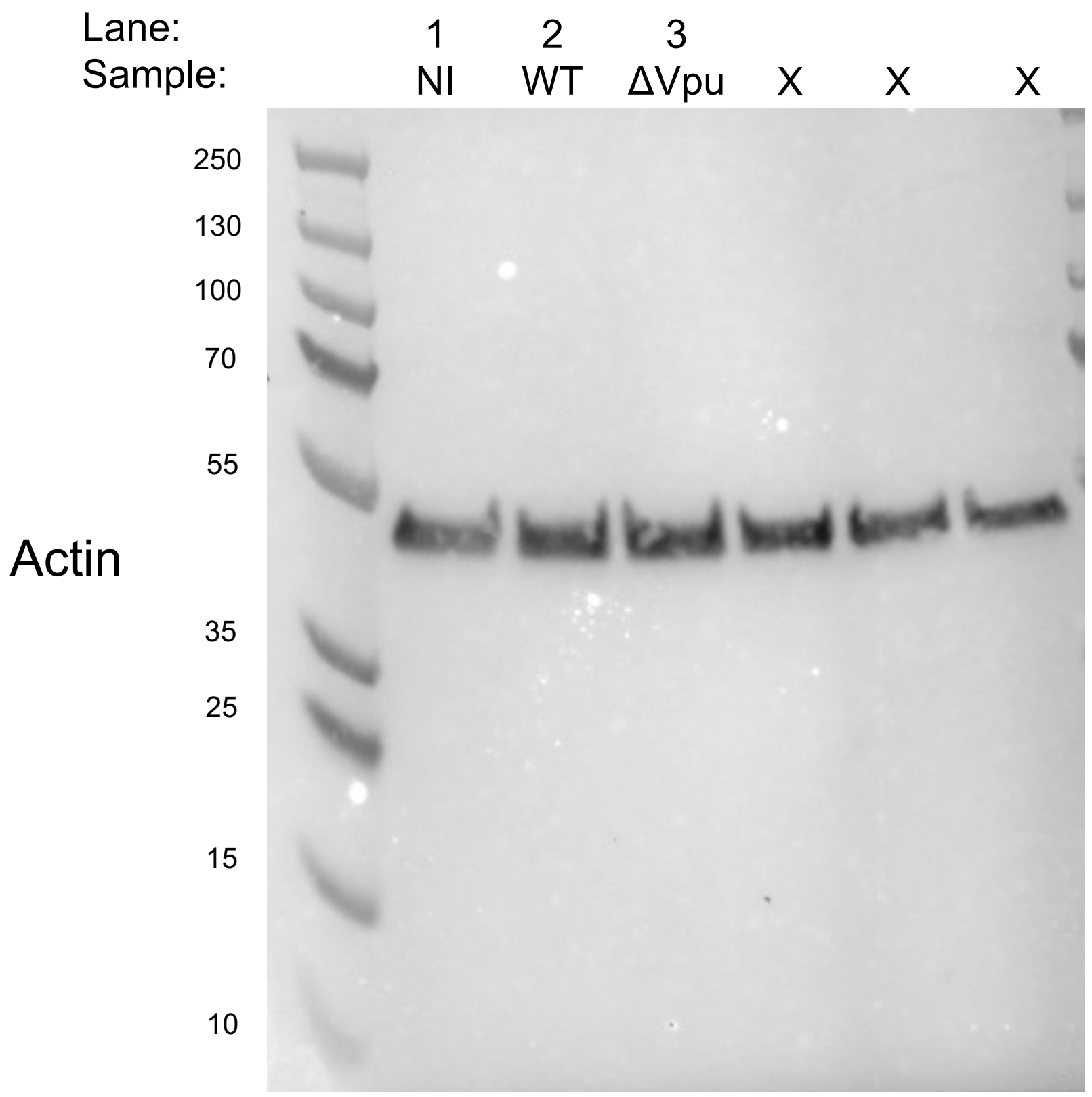

Detected protein: Actin
Membrane: PVDF
Detection method: ECL (ChemiDoc MP)
Exposure time: 3s

X=lane not included in final figure

Original uncropped and unadjusted image corresponding to Figure S4A

BST2

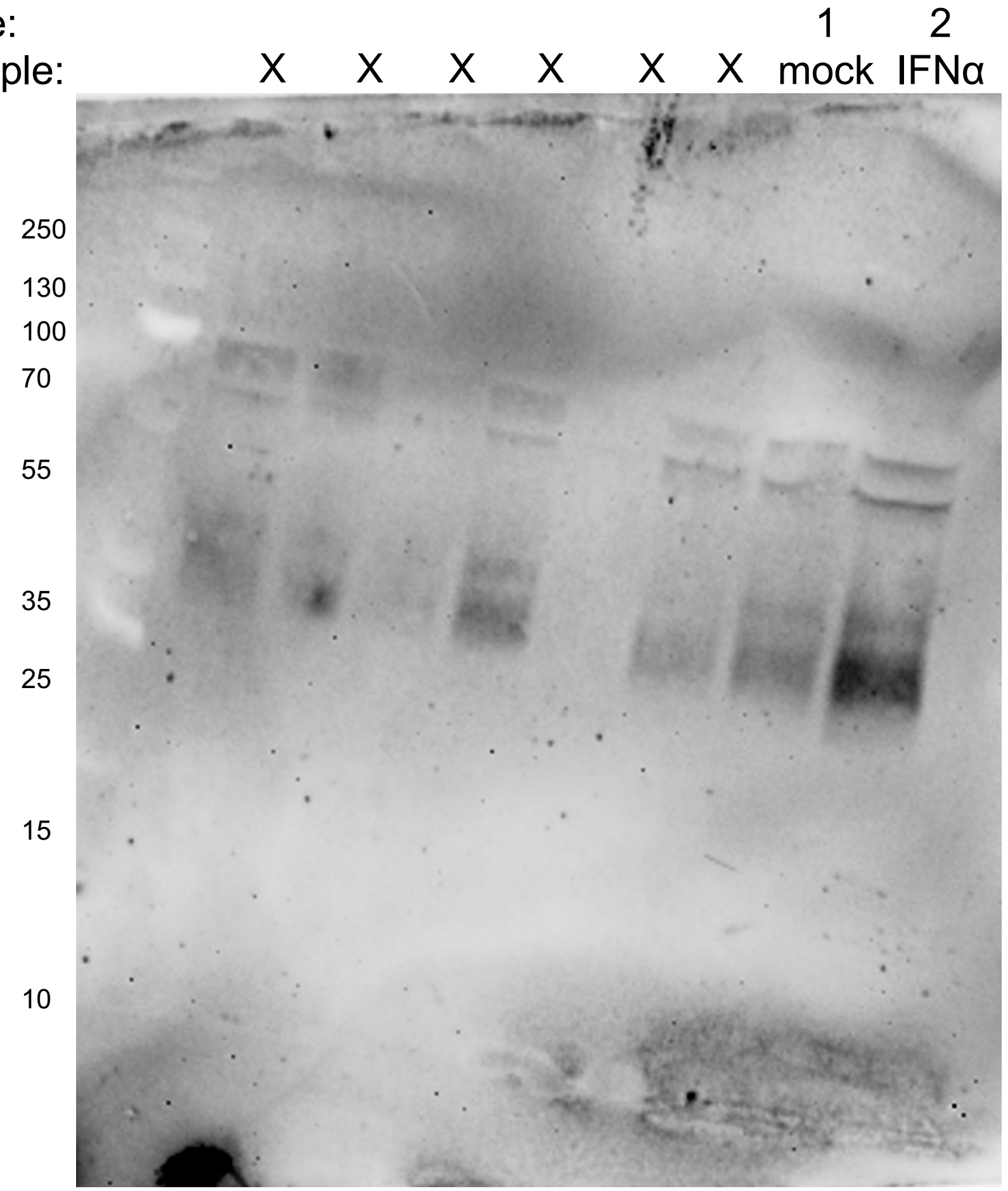

Detected protein: BST2
Membrane: PVDF
Detection method: ECL (ChemiDoc MP)
Exposure time: 180s

Actin

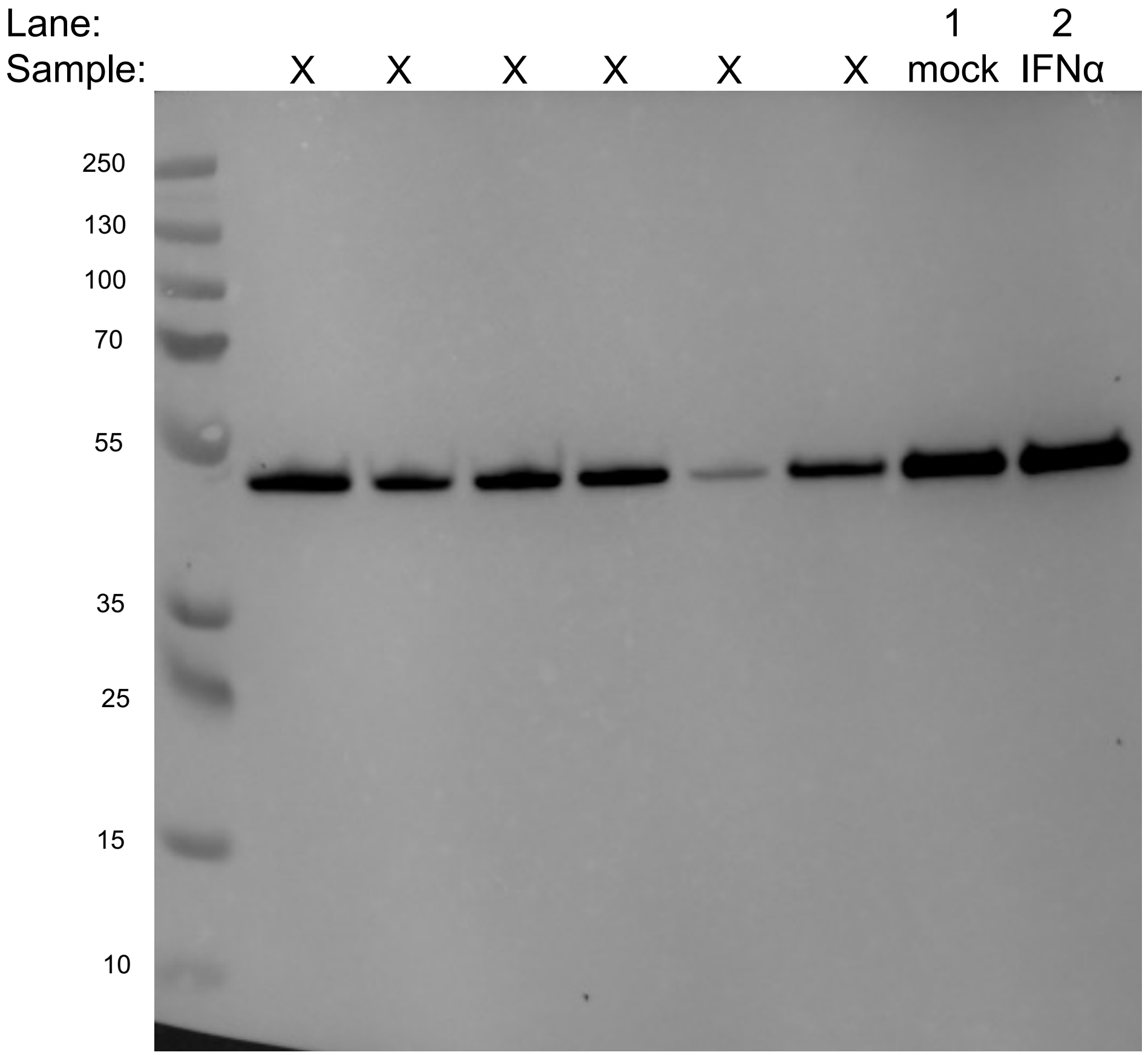

Detected protein: Actin
Membrane: PVDF
Detection method: ECL (ChemiDoc MP)
Exposure time: 3s

X=lane not included in final figure

Original uncropped and unadjusted image corresponding to Figure S4B

BST2

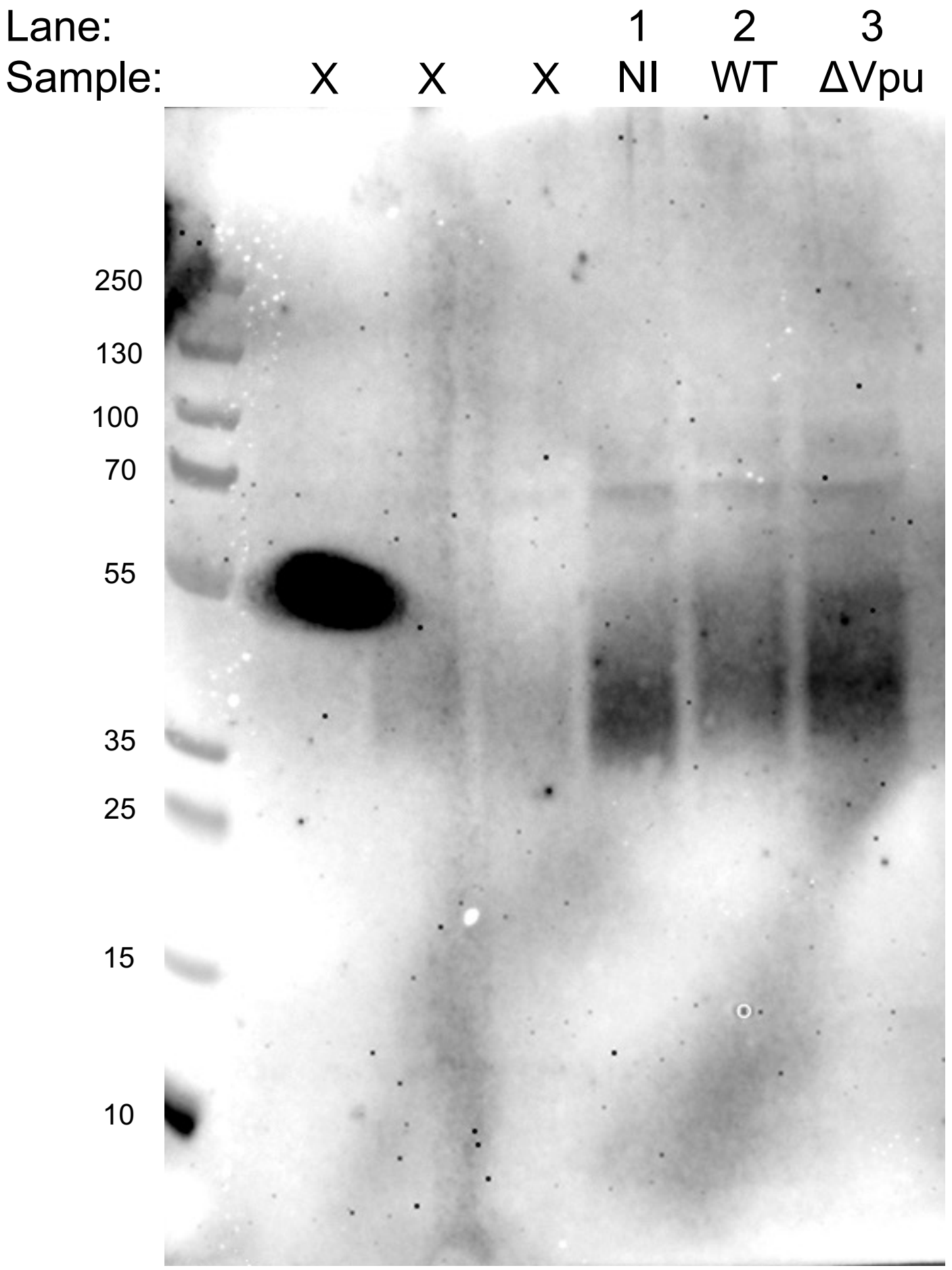

Detected protein: BST2
Membrane: PVDF
Detection method: ECL (ChemiDoc MP)
Exposure time: 180s

Actin

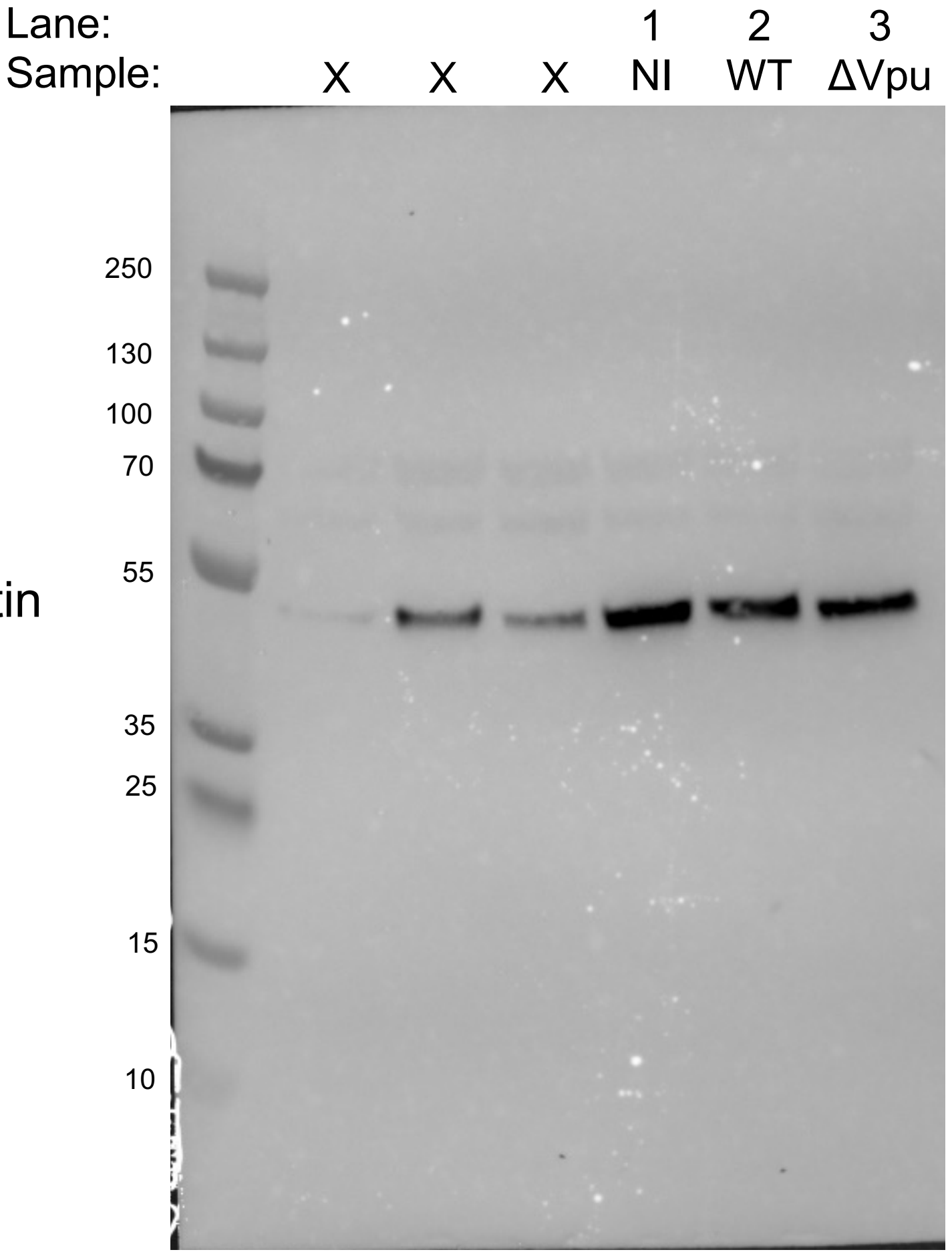

Detected protein: Actin
Membrane: PVDF
Detection method: ECL (ChemiDoc MP)
Exposure time: 3s

X=lane not included in final figure

Original uncropped and unadjusted image corresponding to Figure S4C

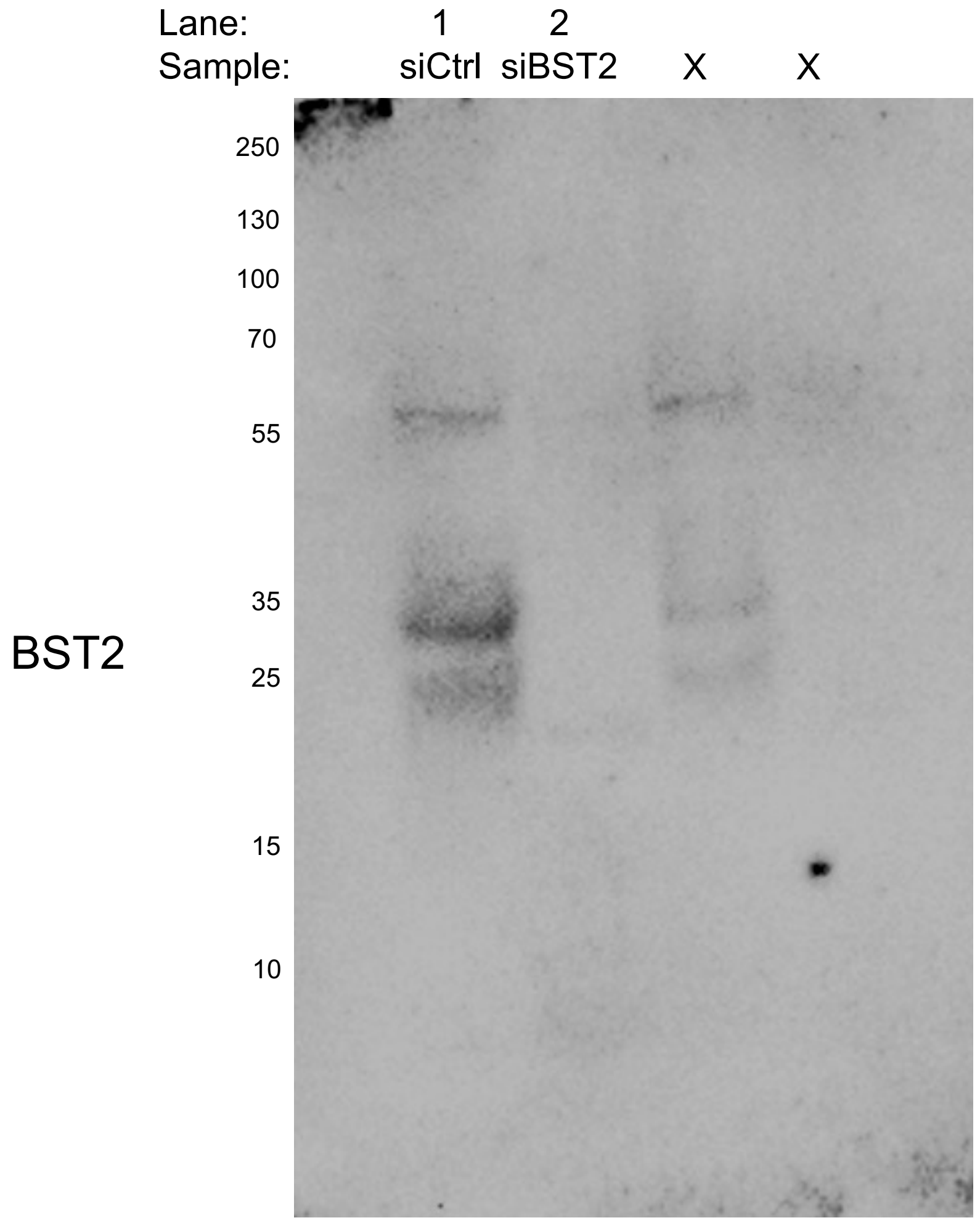

Detected protein: BST2
Membrane: PVDF
Detection method: ECL (ChemiDoc MP)
Exposure time: 180s

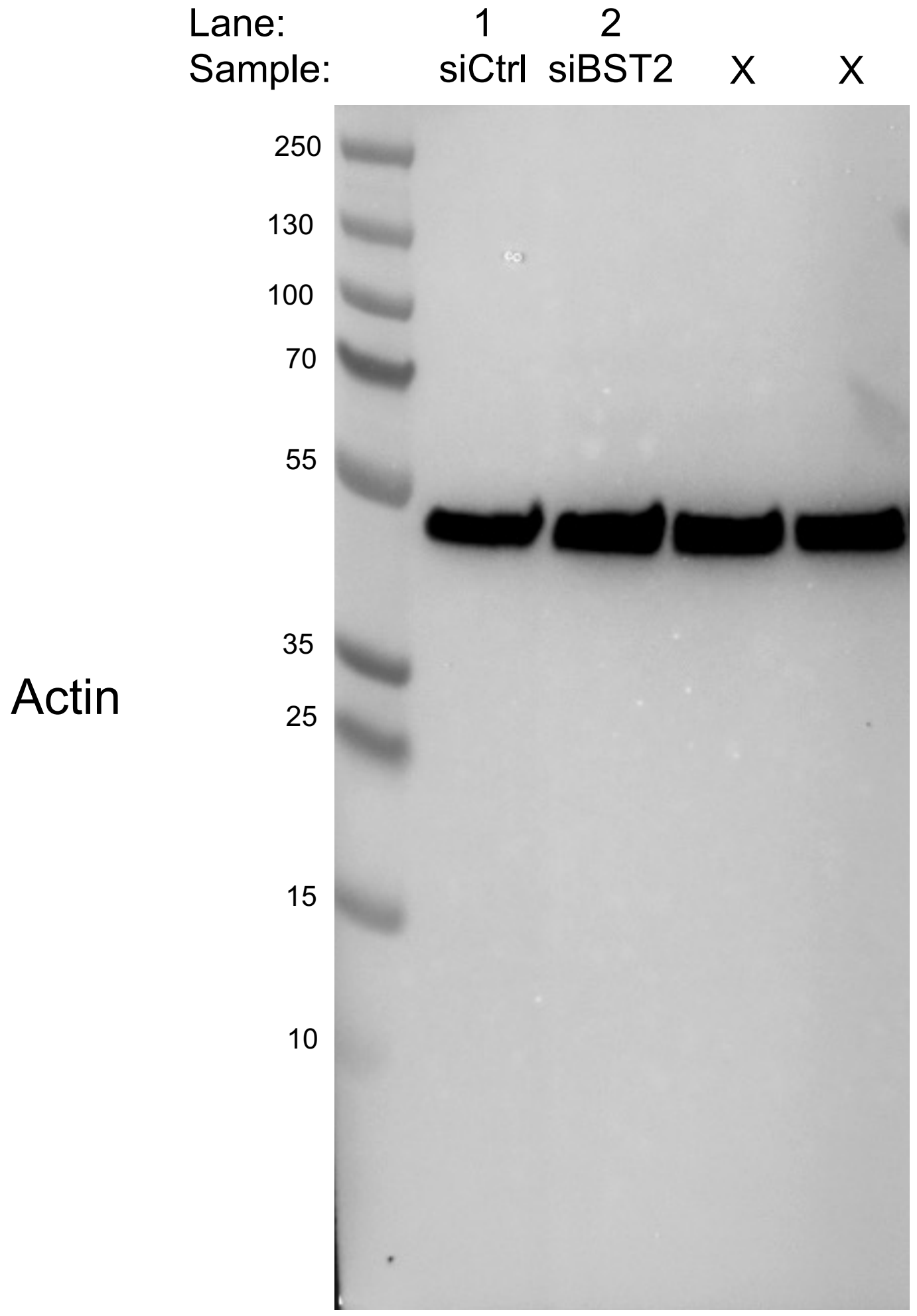

Detected protein: Actin
Membrane: PVDF
Detection method: ECL (ChemiDoc MP)
Exposure time: 3s

X=lane not included in final figure
